# Unveiling the cellular landscape: insights from single-cell RNA sequencing in multiple myeloma

**DOI:** 10.3389/fimmu.2024.1458638

**Published:** 2024-08-30

**Authors:** Xinhan Li, Zhiheng Lin, Fu Zhao, Tianjiao Huang, Weisen Fan, Lijun Cen, Jun Ma

**Affiliations:** ^1^ Shandong University of Traditional Chinese Medicine, Jinan, Shandong, China; ^2^ The First School of Clinical Medicine, Heilongjiang University of Traditional Chinese Medicine, Harbin, China; ^3^ Key Laboratory of Molecular Pathology in Tumors of Guangxi, Department of Transfusion Medicine, Affiliated Hospital of Youjiang Medical University for Nationalities, Baise, Guangxi, China; ^4^ Department of Cardiology, Yantai Hospital of Traditional Chinese Medicine, Yantai, China

**Keywords:** scRNA-seq, multiple myeloma, TFs, pseudotime trajectory, MMP, TIMP

## Abstract

**Objective:**

The aim of this research was to gain a thorough understanding of the processes involved in cell communication and discover potential indicators for treating multiple myeloma (MM) through the use of single-cell RNA sequencing (scRNA-seq). And explored the expression of multiple myeloma-related subgroups on metal ion-related pathways to explore the relationship between MM and metal ions.

**Methods:**

We performed a fair examination using single-cell RNA sequencing on 32 bone marrow specimens collected from 22 individuals at different points of MM advancement and 9 individuals without any health issues. To analyze the scRNA-seq data, we employed advanced computational algorithms, including Slingshot, Monocle2, and other methodologies. Specifically, Slingshot and Monocle2 enabled us to simulate the biological functionalities of different cell populations and map trajectories of cell developmental pathways. Additionally, we utilized the UMAP algorithm, a powerful dimension reduction technique, to cluster cells and identify genes that were differentially expressed across clusters.

**Results:**

Our study revealed distinct gene expression patterns and molecular pathways within each patient, which exhibited associations with disease progression. The analysis provided insights into the tumor microenvironment (TME), intra- and inter-patient heterogeneity, and cell-cell interactions mediated by ligand-receptor signaling. And found that multiple myeloma-related subgroups were expressed higher levels in MMP and TIMP pathways, there were some associations.

**Conclusion:**

Our study presents a fresh perspective for future research endeavors and clinical interventions in the field of MM. The identified gene expression patterns and molecular pathways hold immense potential as therapeutic targets for the treatment of multiple myeloma. The utilization of scRNA-seq technology has significantly contributed to a more precise understanding of the complex cellular processes and interactions within MM. Through these advancements, we are now better equipped to unravel the underlying mechanisms driving the development and progression of this complex disease.

## Introduction

Multiple myeloma (MM) is a type of cancer that affects plasma cells (PCs) and primarily manifests in the bone marrow (BM). It constitutes over 10 percent of all hematologic malignancies (1). MM usually develops from precursor conditions called monoclonal gammopathy of undetermined significance (MGUS) and smoldering multiple myeloma (SMM) (2).

Immunodeficiency characterizes multiple myeloma, a malignant disease that cannot be cured. In comparison to other blood cancers, this one has a gradual beginning. Patients typically show signs of monoclonal gammopathy of uncertain significance (MGUS) at the beginning of their clinical presentation, characterized by the presence of localized myeloma cells and indicating a pre-cancerous state (3). Afterward, the illness advances to smoldering multiple myeloma (SMM) without harming essential organs (4, 5). Ultimately, patients advance to multiple myeloma, displaying clinical symptoms of end-organ dysfunction (6). Continual work is being done to advance immunotherapy options for multiple myeloma, including monoclonal antibodies, bispecific T cell engagers, antibody-drug conjugates, and adoptive cell therapies like CAR-T, CAR-natural killer (NK), and TCR-T (7, 8). Despite some success with these methods, multiple myeloma continues to be incurable because of the development of both natural and acquired resistance to treatment. Hence, it is imperative to investigate new therapeutic possibilities for individuals suffering from this ailment.

The use of single-cell transcriptome analysis has become essential for studying complex biological processes in diverse cell populations (9). Monocle2 algorithm uses the single-cell transcriptome expression matrix to model the biological functions of cell populations. It achieves this by employing unsupervised learning techniques to delineate distinct branches of cell developmental trajectories (10). Additionally, the UMAP algorithm enables the clustering of cells, facilitating the identification of differentially expressed genes across various cellular states. This analysis aids in the identification of pivotal genes that influence diverse differentiation pathways (11).

Traditional methods of bulk RNA sequencing require the analysis of a combination of all cells, which may hide differences in the typical transcriptome of particular cell types. On the other hand, scRNA-seq allows for the analysis of gene expression at a single-cell level, revealing cell-to-cell communication pathways and facilitating the discovery of unique cellular conditions in tumors. Single-cell RNA sequencing offers a more accurate comprehension of the tumor microenvironment, revealing the reasons behind variations within and between patients, along with the communication between cells through ligand-receptor signaling (12). To date, numerous studies employing scRNA-seq have investigated the expression profiles of single cells within bone marrow tissues of multiple myeloma (MM) patients, shedding light on tumor cells and TME cellular components (13–22). In previous studies, the microenvironment of multiple myeloma after lymphodepletion was explored by scRNA-seq, which proved that scRNA-seq has a good role in this regard (23). However, the precise mechanisms governing cell-cell interactions between tumors and the TME in MM remain elusive.

Matrix metalloproteinases (MMPs) and tissue inhibitor of metalloproteinases (TIMPs) play a vital role in the pathogenesis of multiple myeloma (MM), especially for tumor invasion and osteolytic osteopathy (24). So, to study the impact of metal ions on multiple myeloma, we can start from these two pathways.

In order to fully understand the processes involved in cell communication and discover possible indicators for treating myeloma, we carried out an impartial study using scRNA-seq. The research included 32 bone marrow specimens collected from 22 individuals at different points of multiple myeloma development, along with 9 donors who were in good health. The objective was to uncover novel targets for myeloma treatment. These discoveries offer fresh perspectives for future research endeavors and clinical interventions in the field of multiple myeloma (25–27).

## Methods

### Data source

A combined 32 bone marrow specimens were obtained from 22 individuals at different phases of multiple myeloma advancement, in addition to 9 samples from donors in good health. The selection of specific bone marrow specimens for our study was based on a careful consideration of the research objectives and the need to capture the diverse stages of MM advancement. We aimed to encompass a comprehensive representation of the disease progression and its associated molecular changes. ScRNA-seq datas came from GEO website (https://www.ncbi.nlm.nih.gov/geo/), with GSE number GSE124310. The samples included: GSM3528753, GSM3528755, GSM3528757, GSM3528759, GSM3528762, GSM3528764, GSM3528767, GSM3528769, GSM3528771, GSM3528773, GSM3528775, GSM3528777, GSM3528779, GSM3528781, GSM3528783, GSM3528785, GSM3528787, GSM3528789, GSM3528791, GSM3528794, GSM3528796, GSM3528798, GSM3528800, GSM3528802, GSM3528804, GSM3528807, GSM3528809, GSM3528810, GSM3528812, GSM3528814, GSM3528816, GSM3528818.

### Processing of scRNA-seq datas

The research involved analyzing the gene expression data with Seurat software (version 4.3.0) to isolate top-quality cells (28). After quality control, the DoubletFinder package was utilized to identify and remove potential doublet cells (29, 30). The remaining cells were then normalized, and the top 2000 hypervariable genes were selected for further analysis. The gene expression data of these genes were standardized.

Standardized gene expression data underwent Principal Component Analysis (PCA) (31). The Harmony method was applied to remove batch effects between samples. For UMAP dimensionality reduction and visualization of gene expression, the initial 30 significant principal components (PCs) were chosen for uniform manifold approximation and projection (UMAP) dimensionality reduction and visualization of gene expression (32–34).

To eliminate poor-quality cells, those with unusually high nFeature and nCount values were eliminated, as well as cells where mitochondrial gene expression made up over 20% of the total expression, and cells with erythrocyte gene expression surpassing 5% of the total expression were also excluded.

The cell clusters obtained from the UMAP analysis were annotated using the CellMarker database, which is a comprehensive resource for cell type annotation based on previous literature (http://xteam.xbio.top/CellMarker/). This annotation process helped identify the cell types present in the dataset.

Moreover, the research investigated the percentage of various cell categories in the data, offering a glimpse into the cellular makeup of the samples under scrutiny.

In general, the preprocessing and analysis of this data enabled the detection of top-notch cells, elimination of batch discrepancies, reduction of dimensions, and annotation of cell types, laying the groundwork for deeper investigation into the diversity and makeup of cells in the setting of multiple myeloma.

### DEGs

The study identified differentially expressed genes (DEGs) (35) for each cell type by utilizing the FindAllMarkers function (36, 37) within the Seurat software package (38, 39). This analysis was performed on the standardized gene expression data. Specifically, genes expressed in more than 25% of the cells within a cluster and with a log fold change (logFC) value greater than 0.25 were selected as potential marker genes for that cluster.

### KEGG and GO analysis

In order to explore the functional consequences of these differentially expressed genes (DEGs), we performed enrichment analyses using KEGG (Kyoto Encyclopedia of Genes and Genomes) (40–44) and GO (Gene Ontology) (45, 46). Genes that had an adjusted p-value below 0.05 were deemed statistically significant in the analysis. ClusterProfiler software (v0.1.1) was used to analyze and enrich cluster-specific biomarker genes (47–49).

The researchers conducted enrichment analyses to understand the biological processes, molecular functions, and pathways linked to the various cell types identified in the study. This data can provide insight into the operational traits and possible functions of particular cell varieties within the framework of multiple myeloma (50).

### Distinguishing between myeloma cells and non-cancerous plasma cells using inferCNV

The researchers in the study sought to differentiate myeloma cells from non-cancerous plasma cells by analyzing the copy number variation (CNV) signal in various genomic regions. The inferCNV package (51) was employed, accessible on GitHub at https://github.com/broadinstitute/inferCNV/wiki.

T-NK cells were utilized as a reference to predict the initial CNV signal, leading the researchers to identify myeloma cells as subgroups with significant copy number variation. By using this method, they were able to pinpoint genetic areas that showed notable changes in copy numbers in myeloma cells when compared to non-cancerous plasma cells.

### Determination of cell subgroups

After extracting all myeloma cells, they were normalized again to identify the top 2000 hypervariable genes. The expression data of these genes were then standardized. Following this, the standardized gene expression data underwent principal component analysis (PCA).

### Trajectory analysis

To investigate the tumorigenesis of myeloma cells, the researchers employed three software packages to analyze the trajectory of myeloma cell subgroups.

The CytoTRACE algorithm was used to evaluate the stemness of cells in each subgroup. Stemness refers to the degree to which a cell retains its ability to differentiate into various cell types. By evaluating the stemness of myeloma cell subgroups, the researchers aimed to gain insights into the differentiation potential and hierarchy within the cell population.

The Monocle software toolkit (52) was employed to reconstruct the trajectory of cell differentiation. Monocle is a well-liked software used for examining data from single-cell RNA sequencing and predicting the paths of development (53). By utilizing Monocle, we aimed to understand the progression of myeloma cells from less differentiated to more differentiated states. The examination may offer understanding into the cellular mechanisms and control systems implicated in myeloma tumor formation. Utilizing the DDRTree technique, the data was condensed to observe the evolution of cell clusters along the novel path. DDRTree is a dimensionality reduction technique that helps reveal the underlying structure and relationships within high-dimensional datasets. By applying DDRTree, we could observe the progression and branching patterns of myeloma cell subgroups in a reduced-dimensional space.

Furthermore, the slingshot method was employed to analyze the cell trajectory during the differentiation of myeloma cells. Slingshot is a computational method that infers cell lineages and estimates the expression levels of each lineage over time. By using slingshot, the researchers aimed to gain insights into the temporal dynamics and lineage relationships of myeloma cell subgroups during the process of differentiation.

### Intercellular interaction analysis

Researchers used the ‘CellChat’ package (version 1.6.1) to study the network of interactions between myeloma subgroups and other cells in the microenvironment (54). The purpose of this package is to examine and deduce communication between cells through signal pathways and interactions between receptors and ligands.

The researchers utilized the CellChat software to investigate the interactions between ligands and receptors of niche cell subtypes (microenvironment cells) and malignant cells (myeloma subgroups). Ligands are signaling molecules secreted by cells, while receptors are proteins on the cell surface that can bind to specific ligands and initiate signaling pathways. By identifying the ligand-receptor pairs between different cell types, the researchers aimed to understand the potential signaling interactions and communication between the microenvironment cells and myeloma subgroups.

Furthermore, the CellChat package allowed the researchers to explore how signal pathways were coordinated among various cell types. This analysis helps in understanding the overall communication network and signaling crosstalk between different cell populations within the myeloma microenvironment. By studying the coordination of signal pathways, the researchers could gain insights into the complex cellular interactions and regulatory mechanisms involved in myeloma tumorigenesis and progression.

### SCENIC analysis

For the research, we employed the pyscenic software (v0.10.0) in Python (v3.7) to build a gene regulatory network and pinpoint consistent cell conditions with scRNA-seq data.

Initially, pySCENIC was utilized to assess the enrichment of transcription factors (TFs) and the effectiveness of regulators (55). This analysis aimed to identify TFs that were enriched in specific cell states and regulators that were active in driving gene expression changes within those states. By assessing TF enrichment and regulator activity, the researchers gained insights into the regulatory mechanisms underlying cell state transitions and gene expression patterns.

We utilized co-expression and DNA motif analysis to build the gene regulatory network. Co-expression analysis identifies genes that are co-regulated and likely to be part of the same regulatory network. DNA motif analysis involves examining the presence of specific DNA sequence motifs, which are binding sites for TFs, in the regulatory regions of genes. The scientists created a gene regulatory network by analyzing co-expression and DNA motifs to deduce the regulatory connections between transcription factors and target genes.

To determine the cell state, we examined the activity of the gene regulatory network in each cell. Through evaluating the behavior of transcription factors and controllers in the system, scientists were able to categorize cells into distinct conditions and pinpoint steady cell conditions by analyzing their gene expression profiles and regulatory behavior. To guide the search for the transcription factor regulatory network around the transcription initiation site, we utilized the ranking of gene motifs within a 10 kb region surrounding the transcription initiation site. This ranking provided a guide for identifying potential TF binding sites and regulatory interactions in the vicinity of gene promoters.

In this study, we employed the pySCENIC software to construct a gene regulatory network and identify consistent cell conditions using scRNA-seq data. They utilized pySCENIC to assess the enrichment of transcription factors (TFs) and regulators, gaining insights into the regulatory mechanisms underlying cell state transitions and gene expression patterns. Co-expression and DNA motif analysis were used to build the gene regulatory network, identifying co-regulated genes and potential TF binding sites. By evaluating the activity of the gene regulatory network, we categorized cells into distinct conditions and pinpointed steady cell conditions based on gene expression profiles and regulatory behavior. The ranking of gene motifs around the transcription initiation site guided the search for potential TF binding sites and regulatory interactions.

Overall, this method played a crucial role in deciphering the gene regulatory landscape and understanding cell states in the study.

## Results

### scRNA sequencing annotated major cell types in multiple myeloma progression

A combined 32 bone marrow specimens were obtained from 22 individuals at different phases of multiple myeloma advancement, in addition to 9 samples from donors in good health. Through the use of scRNA-seq, we were able to pinpoint the primary cell categories contributing to the advancement of multiple myeloma.

**Figure 1:**
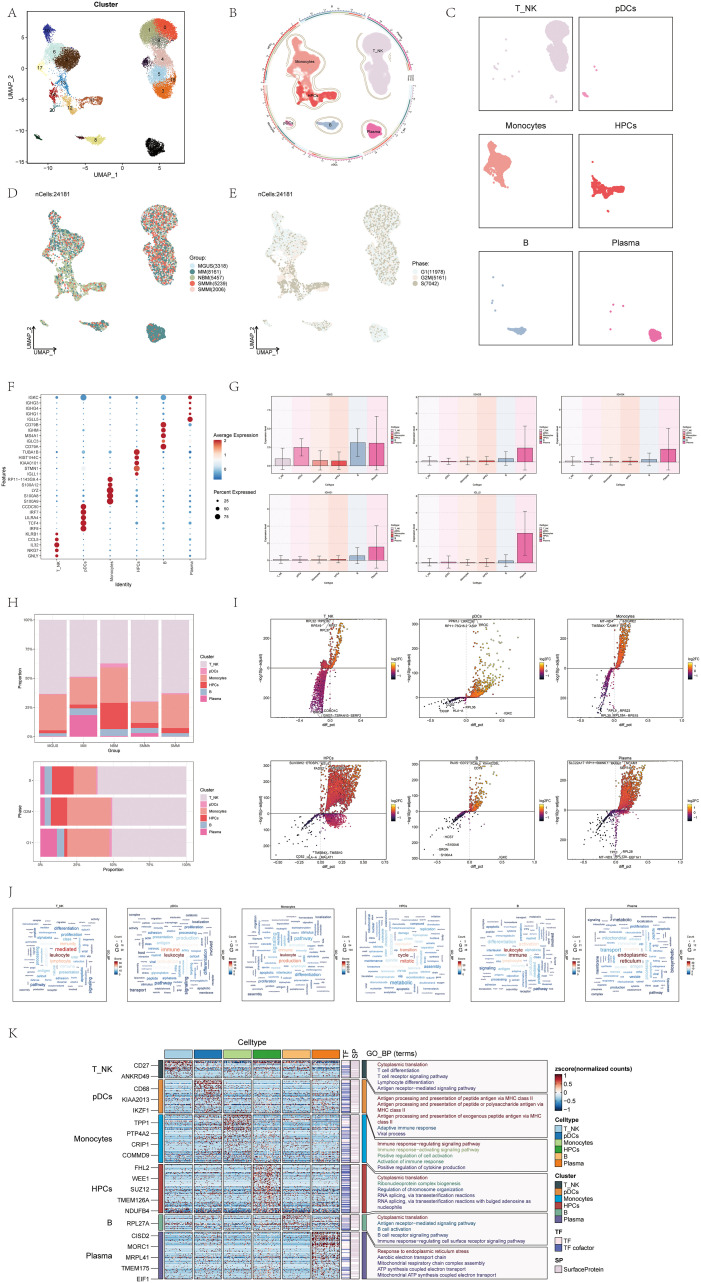
scRNA sequencing revealed major cell types during the progression of multiple myeloma (MM). **(A)** UMAP plot showed 21 clusters of cells from multiple myeloma patients. Each point represented an individual cell colored according to the cell cluster. **(B)** Chord diagram displayed different cell types (T_NK cells, Monocytes cells, HPCs, pDCs, Plasma cells, B cells) within multiple myeloma cells. Different colors represented different cell types. **(C)** Facet plots showed the distribution of cell clusters corresponding to six different cell types in multiple myeloma. **(D)** UMAP plot displayed the distribution of five different groups corresponding to the 21 cell clusters in multiple myeloma patients. Different colors represented different groups. **(E)** UMAP plot illustrated how phases were distributed among the six distinct cell types in cases of multiple myeloma. **(F)** Dot plot displayed the differential expression of Top5maker genes in the six different cell types of multiple myeloma. The dots’ size indicated the proportion of gene expression in the subgroups, while the intensity of color indicated the genes’ expression level. **(G)** Box plots showed the expression of Top5maker genes in Plasma cells. **(H)** Bar plots showed the proportions of the six different cell types in different groups (above) and different phases (below) in multiple myeloma. Different colors represented different cell types. **(I)** Volcano plots illustrated the gene expression differences in the six distinct cell types of multiple myeloma. **(J)** Word cloud plots showed the enrichment of gene pathways in the six different cell types of multiple myeloma. The size of the letters represents the number of enriched pathways, and the color represents the enrichment scores of the pathways in different cell subgroups. **(K)** GO-BP enrichment analysis revealed the biological processes linked to the six distinct cell types found in multiple myeloma.

After performing initial quality control measures and eliminating batch effects, we retained a total of 24,181 cells for further analysis. These cells were categorized into 21 distinct clusters, each assigned a different color (**Figure 1A**). By analyzing the distinct gene expression patterns in these groups, we categorized them into six cell types (56): Monocytes (clusters 2, 6, 10, 13, 17), Hematopoietic Progenitor Cells (HPCs) (clusters 11, 12, 14, 20), Plasmacytoid Dendritic Cells (pDCs) (cluster 18), B cells (clusters 8, 19), T_NK cells (clusters 0, 1, 3, 4, 5, 9, 15, 16), and Plasma cells (cluster 7) (**Figure 1B**).

For visualizing the distinct cellular shapes of each type of cell, we utilized Uniform Manifold Approximation and Projection (UMAP) to reduce dimensionality (**Figure 1C**). Additionally, we utilized UMAP to illustrate the distribution of the 24,181 cells across different sample groups and cell cycle phases (**Figures 1D, E**).

**Figure 1F** displayed a dot plot showing the top 5 genes with high expression levels in different cell types. As MM is a type of blood cancer defined by the existence of unusual plasma cells in the boneEI marrow, we examined the average expression level of five genes (IGKC, IGHG3, IGHG4, IGHG1, IGLL5) in the six cell types and illustrated their expression patterns in **Figure 1G**. Interestingly, these five genes exhibited predominantly elevated expression in plasma cells, aligning with the characteristic abnormal plasma cells observed in multiple myeloma, which underwent monoclonal immunoglobulin (IG) malignant proliferation in the bone marrow (57, 58).

We used a histogram to demonstrate how the six cell types are distributed among various groups and stages. Notably, T_NK cells constituted the lowest proportion in the normal bone marrow (NBM) group, but their proportion increased in all other groups. Conversely, plasma cells accounted for a larger proportion in multiple myeloma (MM) samples and the G1 cell cycle phase (**Figure 1H**).

To describe the differentially expressed genes and biological processes among the six cell types, we utilized volcano diagrams (**Figure 1I**) and word cloud diagrams (**Figure 1J**), respectively. The volcano diagrams could detail five gene types that were highly expressed in each cell type. The word cloud plots described in detail the biological processes with high expression in each cell type, among which the biological process with the highest correlation with these three cell types (T_NK cells,pDCs,Monocytes) was leukocyte, the biological process with the highest correlation with HPCs was cycle, the biological process with the highest correlation with B cells was immune, and the biological processes with the highest correlation with plasma cells were endoplasmic, reticulum. Furthermore, a heatmap was utilized to display the outcomes of gene ontology biological process (GO-BP) enrichment analysis for the varying gene expression in the six cell types (**Figure 1K**).

### Identification of multiple myeloma cell subtypes

To gain a deeper insight into the features of plasma cells in multiple myeloma, we utilized inferCNV (**Supplementary Figure 1**) for the analysis of single-cell RNA-seq data derived from multiple myeloma cells. This allowed us to discern myeloma cells and conduct additional subclustering. As a result, we successfully clustered a total of 1,488 multiple myeloma cells into four distinct cell subgroups.

**Figure 2:**
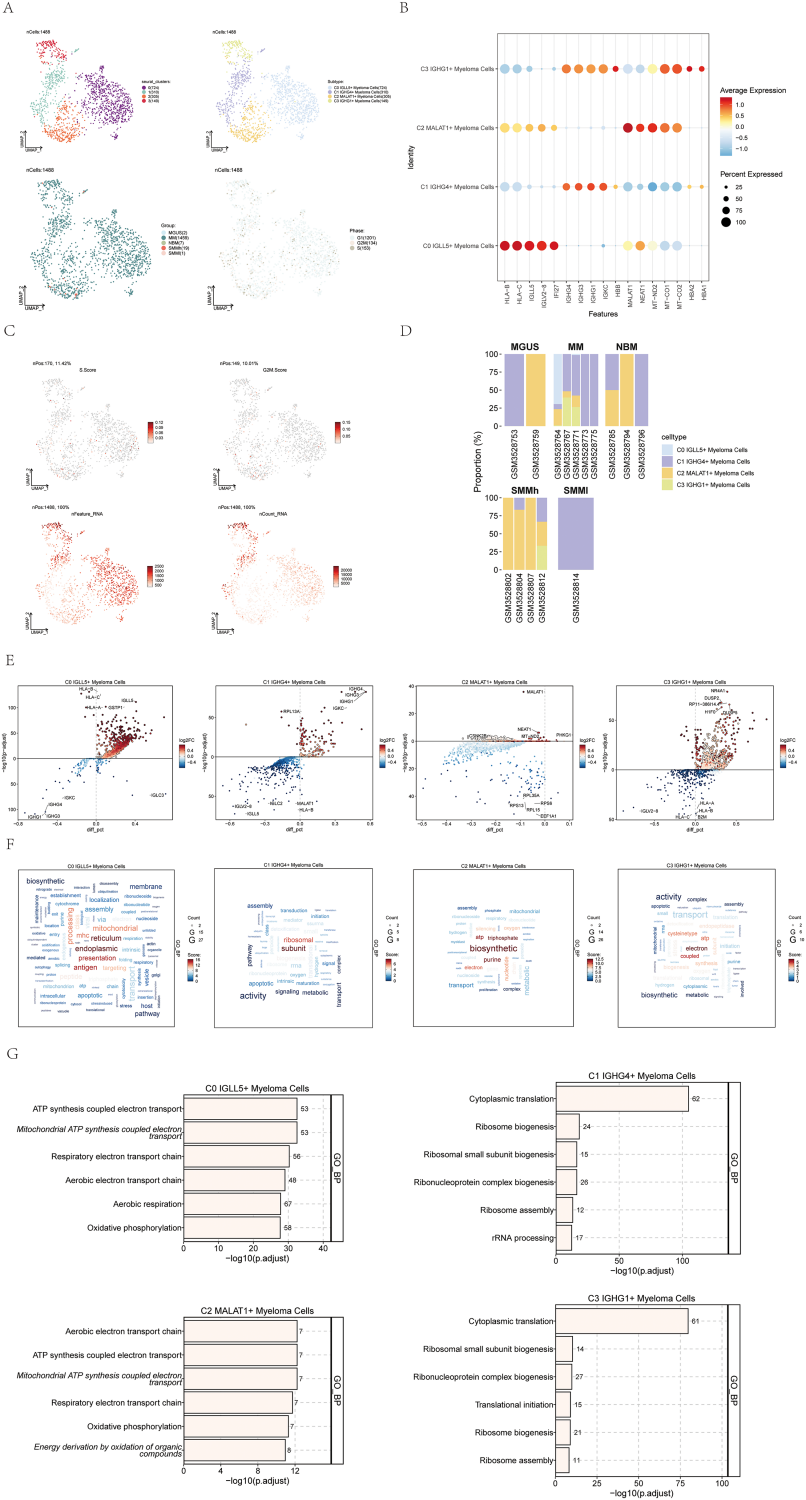
Visualization of multiple myeloma cell subpopulations. **(A)** UMAP visualization displayed four distinct groupings of myeloma cells from MM patients along with the cell count in each group (top left); another UMAP visualization depicted the cell subgroups associated with the four groupings (top right); additionally, a UMAP plot illustrated the arrangement of categories linked to the four cell subgroups (bottom left); lastly, a UMAP plot showcased the spread of stages connected to the four cell subgroups (bottom right). Each point represented an individual cell colored according to the cell subpopulation. **(B)** A dot plot was used to show the variation in expression levels of the top 5 genes in the four different cell subgroups. Dot size indicated gene expression percentage in subgroups, while color intensity indicated gene expression level. **(C)** UMAP plots displayed the important characteristics of the four cell subgroups: S.score, G2M.score, nFeature_RNA, nCount_RNA. **(D)** Bar graphs displayed the distribution of the four cellular subgroups in every sample across the five categories the groups represented. **(E)** Volcano plots exhibited the gene expression of differentially expressed genes across the four cell subgroups. **(F)** Gene pathway enrichment in the four cell subpopulations was demonstrated through word cloud plots. The size of the letters represented the number of enriched pathways, and the color represented the enrichment scores of the pathways in different cell subpopulations. **(G)** The GO-BP enrichment analysis revealed the biological processes linked to the four cell subgroups.

The four identified cell subgroups were as follows: C0 IGLL5+ Myeloma Cells (724 cells), C1 IGHG4+ Myeloma Cells (310 cells), C2 MALAT1+ Myeloma Cells (305 cells), and C3 IGHG1+ Myeloma Cells (149 cells). We visualized the distribution of these four cell subgroups across sample groups and cell cycle phases, as depicted in **Figure 2A**.

To highlight the highly expressed genes specific to each of the four cell subtypes in multiple myeloma, we utilized a dot plot, showcasing the top 5 genes for each subtype (**Figure 2B**). Moreover, we visualized several relevant features of these four cell subgroups, including S.score, G2M.score, nFeature_RNA, and nCount_RNA, as shown in **Figure 2C**.

The distribution of the four cell subgroups across different sample groups was demonstrated using histograms in **Figure 2D**. Notably, we observed that C0 IGLL5+ Myeloma cells were only present in two patients within the multiple myeloma (MM) group.

To fully grasp the variations in gene expression and biological functions within the four cell subgroups, we utilized volcano plots (**Figure 2E**) and word cloud plots visualizations (**Figure 2F**). Among them, the biological processes with the highest correlation with C0 IGLL5+ Myeloma Cells were endoplasmic and reticulum. The biological process with the highest correlation with C1 IGHG4+ Myeloma Cells was subunit; The biological process most associated with C2 MALAT1+ Myeloma Cells was biosynthetic; The biological process most associated with C3 IGHG1+ Myeloma Cells was electron. Additionally, an analysis was performed to enrich the gene ontology biological process (GO-BP) for the genes that were expressed differently in the four cell subgroups, and the findings were visualized in **Figure 2G** using a heatmap.

### Visualization of a pseudotime analysis of multiple myeloma cells by CytoTRACE and monocle

**Figure 3:**
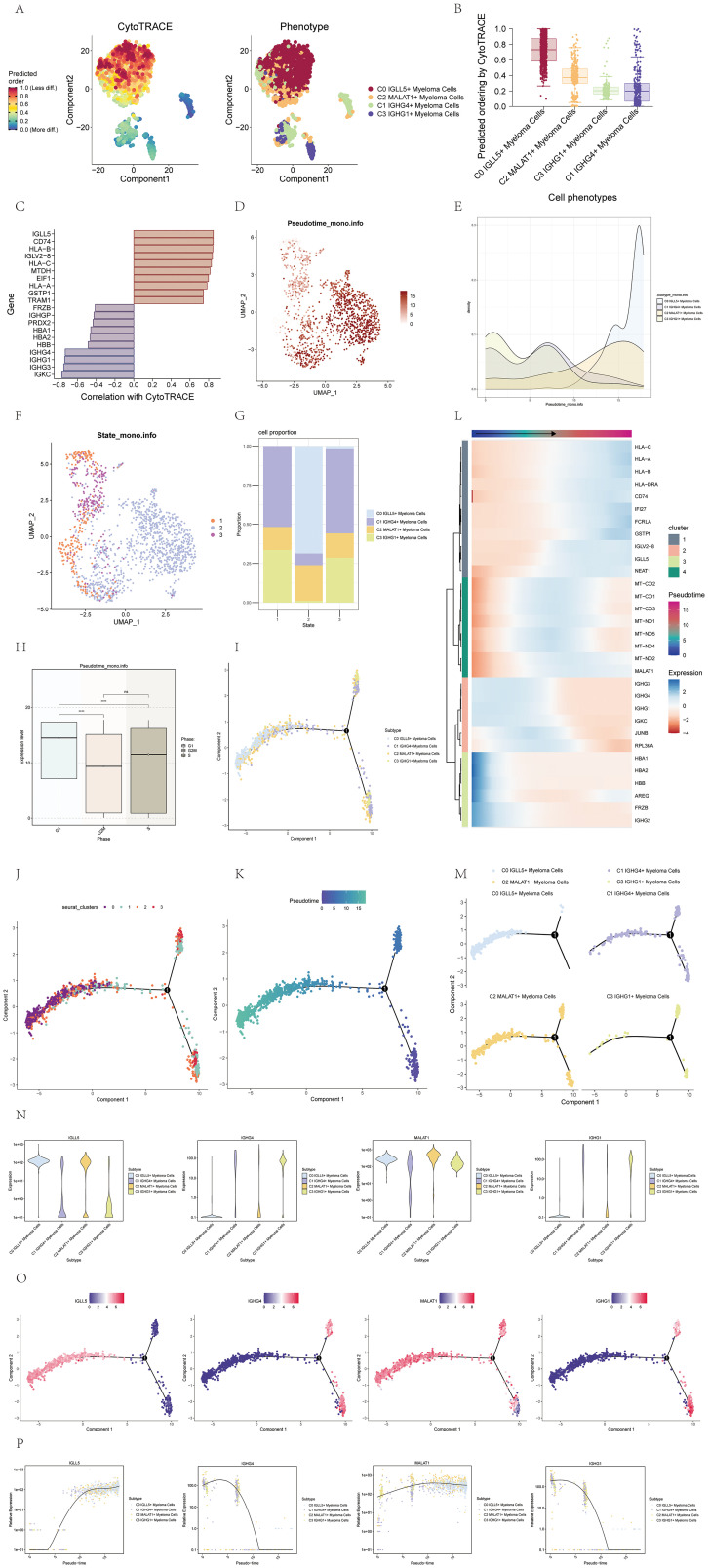
Visualization of pseudotime analysis of multiple myeloma cells using CytoTRACE and monocle. **(A)** The differentiation status analysis of multiple myeloma cells was shown in a 2D plot on the left using CytoTRACE. Colors indicated the degree of differentiation. The CytoTRACE results for various subpopulations of multiple myeloma cells were displayed in the plot on the right. Various colors indicated distinct groups of cells. **(B)** Box plot showed the predicted ordering of cell subpopulations by CytoTRACE. **(C)** Bar plot showed genes correlated with the highest and lowest differentiation levels based on their correlation with CytoTRACE. **(D)** The pseudotime distribution of cell subpopulations in multiple myeloma was visualized on a UMAP plot. **(E)** Ridge plot displayed the pseudotime distribution of cell subpopulations in multiple myeloma. **(F)** UMAP plot illustrated the distribution of pseudotime for cell subpopulations in multiple myeloma. **(G)** A bar graph displayed the percentages of the four cellular subgroups in various stages throughout the pseudotime. **(H)** Box plot displayed the expression patterns of different phases along the pseudotime. *, p ≤ 0.05; **, p<0.01; ***, p<0.001; ****, p<0.0001 indicated significant differences; ns indicated no significant difference. **(I)** The UMAP visualization depicted how the four cell subpopulations were distributed across pseudotime. **(J)** UMAP plot displayed the distribution of the four cell clusters along the pseudotime. **(K)** UMAP plot displayed the pseudotime trajectory of multiple myeloma cells. **(L)** Expression patterns of multiple myeloma-related genes along the pseudotime were shown. The x-axis represented the pseudotime order, and the y-axis represented the average expression level of each gene in the current cell state. **(M)** Facet plots displayed how the four cell subpopulations were distributed across the pseudotime. **(N)** Violin plots showed how genes were expressed in the four cell subpopulations over pseudotime. **(O)** UMAP visualizations showed how named genes were distributed across the four cell subpopulations along the pseudotime. **(P)** Scatter plots displayed the variations in gene expression of identified genes in four cellular subgroups across the pseudotime.

To elucidate the differentiation and developmental relationship among the four cell subgroups of multiple myeloma cells, we conducted an analysis of cell differentiation using CytoTRACE (**Figure 3A**). The results were visualized, and it became evident that the four cell subgroups exhibited a differentiation pattern from C0 to C2, C3, and finally C1 (**Figure 3B**). Specifically, the C0 cell subgroup displayed the highest degree of cell stemness, indicating its primitive nature in the differentiation hierarchy.

To further investigate the genes associated with the most and least differentiated cells, we utilized a bar chart that displayed these genes based on their correlation with CytoTRACE (**Figure 3C**). This visualization allowed us to identify the genes that exhibited the strongest and weakest associations with the differentiation process.

To visualize the distribution of the four cell subgroups along the pseudotime series, we employed UMAP and ridge plots. Notably, the C0 IGLL5+ Myeloma cells were found to be positioned towards the end of the pseudotime series (**Figures 3D, E**).

To provide a comprehensive depiction of the cell subgroup states along the pseudotime series, we utilized UMAP plots and histograms. Based on these visualizations, we identified three distinct states. Specifically, the C0 IGLL5+ Myeloma Cells were primarily located in state 2, while the C1 IGHG4+ Myeloma Cells were predominantly distributed across state 1 and state 3. The C2 MALAT1+ Myeloma Cells exhibited distribution across all three states, whereas the C3 IGHG1+ Myeloma cells were mainly found in state 1 and state 3 (**Figures 3F, G**).

To explore the relationship between the pseudotime sequence and the cell cycle phase, we employed box plots. The findings indicated that the levels of expression in the four cell subgroups were elevated during the G1 phase in contrast to the G2M and S phases, with statistical significance (p<0.01) (**Figure 3H**).

UMAP plots were further utilized to showcase the distribution of the four cell subgroups along the pseudotime sequence. The cell subgroups exhibited differentiation from the two branches on the right side towards the left side, converging at the first branch point, and continuing to differentiate towards the left. Most cells of the C3 subgroup were located at the beginning of the pseudotime series, while the majority of cells in the C0 subgroup were positioned at the end of the pseudotime series. The C1 and C2 subgroups displayed distribution at various points along the pseudotime series (**Figures 3I–K**). The heatmap and facet plots of the top genes for each subgroup along the pseudotime series further supported these observations (**Figures 3L, M**).

To illustrate the distribution of specific genes along the pseudotime sequence of the four cell subgroups, we employed violin plots, UMAP plots, and pseudotime scatter plots (**Figures 3N–P**). These visualizations allowed us to examine the expression patterns and dynamics of the named genes within each cell subgroup throughout the pseudotime progression. By analyzing the violin plots, we could observe the distribution and variation in gene expression levels across the pseudotime series. The UMAP plots provided a spatial representation of the gene expression patterns within the cell subgroups, enabling us to identify any spatial clustering or dispersion of cells based on gene expression. The pseudotime scatter plots showcased the gene expression levels of individual cells within each subgroup along the pseudotime axis, providing insights into the temporal dynamics of gene expression changes during cell differentiation. Collectively, these visualizations facilitated a comprehensive understanding of how the named genes were expressed and regulated within the pseudotime sequence of the four cell subgroups.

### Pseudotime trajectory slingshot analysis of multiple myeloma cell subgroups

**Figure 4:**
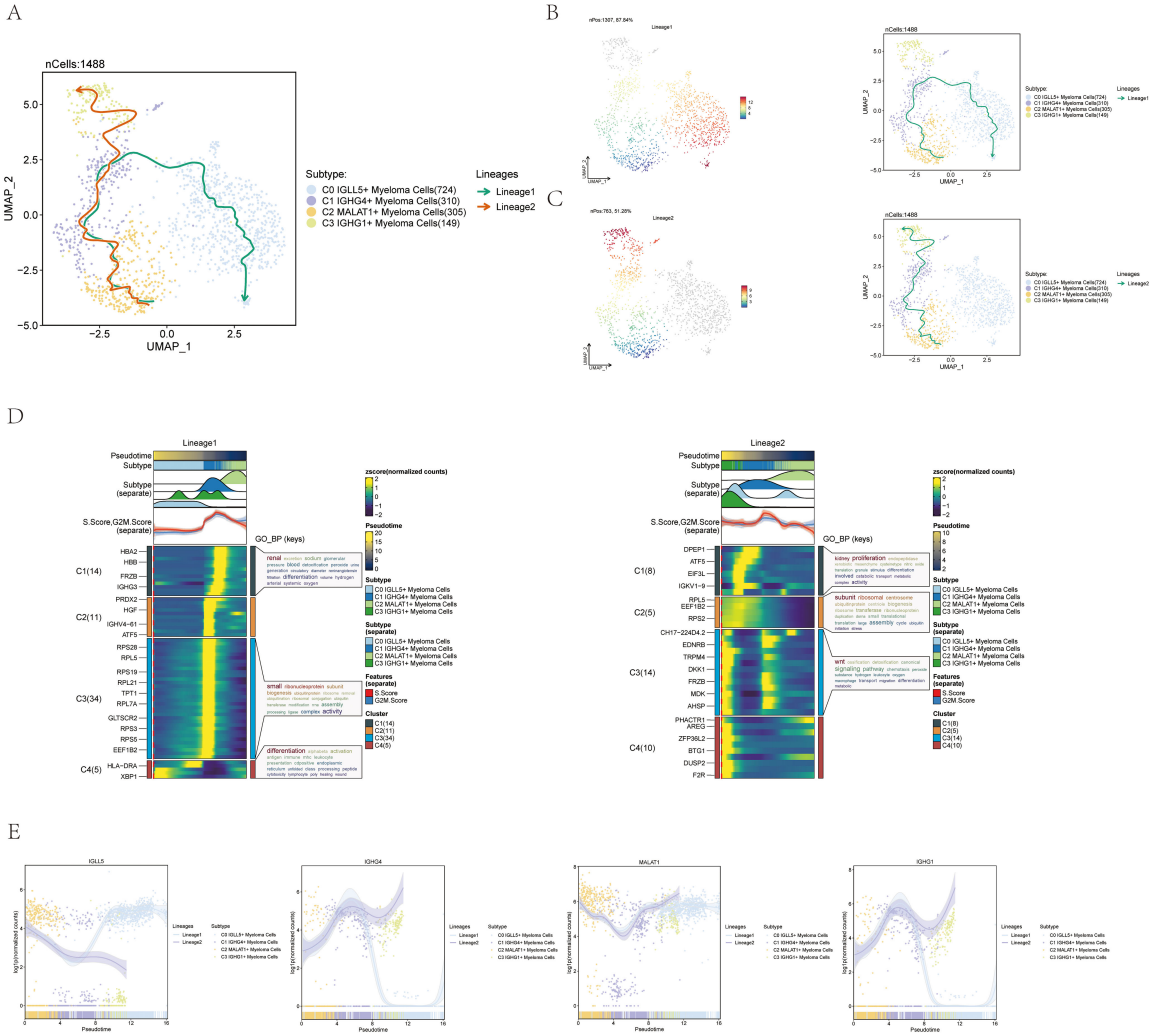
Pseudotime trajectory analysis of multiple myeloma cell subpopulations using slingshot. **(A)** UMAP visualization displayed the spread of two distinct paths of development modeled by pseudotime in every cell of multiple myeloma. **(B)** UMAP plot displayed Lineage1’s changes over pseudotime on the left and its differentiation trajectory over pseudotime on the right. **(C)** UMAP plot displayed Lineage2’s changes over pseudotime on the left and its differentiation trajectory on the right. **(D)** The analysis of GO-BP enrichment revealed the biological processes associated with the two pseudotime paths of various subpopulations of multiple myeloma cells. Left: Lineage1; Right: Lineage2. **(E)** Scatter plots displayed the paths of identified genes in the four subsets of multiple myeloma cells across the two lineages derived from slingshot visualization.

To investigate the presence of continuous branching lineage structures in multiple myeloma cells, we utilized slingshot to analyze the pseudotime trajectories of the four cell subgroups. As a result, two lineages, namely lineage1 and lineage2, were identified (**Figure 4A**).

To visualize the distribution and trajectory of each lineage, UMAP diagrams were employed. The differentiation end of lineage1 was represented by the C0 subgroup, while the differentiation end of lineage2 was represented by the C3 subgroup (**Figures 4B, C**). These diagrams provided a spatial representation of the two lineages and demonstrated their distinct trajectories along the pseudotime axis.

Additionally, a GO-BP enrichment analysis was conducted to understand the biological processes linked to each lineage. In lineage1, the C1 subgroup was found to be related to renal-related biological processes, while the C3 subgroup was associated with small-related biological processes. The C4 subgroup was linked to differentiation-related biological processes, and the C1 and C2 subgroups were associated with proliferation and subunit-related biological processes, respectively. In lineage2, the C3 subgroup was found to be related to Wnt-related biological processes (**Figure 4D**).

To further explore the expression patterns of named genes within different subgroups along lineage1 and lineage2, scatter plots were utilized. The graphs displayed the spread of identified genes among the subcategories in each lineage and illustrated the changes in differentiation over the pseudotime series (**Figure 4E**). It was seen that C0 IGLL5+ Myeloma Cells was expressed higher at the end of lineage1. The visual representations offered important understandings on how the named genes are expressed and regulated in lineage1 and lineage2 within multiple myeloma cells.

### CellChat analysis between cells

To comprehensively investigate complex cellular responses and understand cell-cell interactions, we conducted an analysis of cell-cell relationships and ligand-receptor communication networks. Initially, we constructed an intercellular communication network that encompassed various cell types, including the four subtypes of multiple myeloma cells, Monocytes, B cells, T_NK cells, Plasma cells, and others.

**Figure 5:**
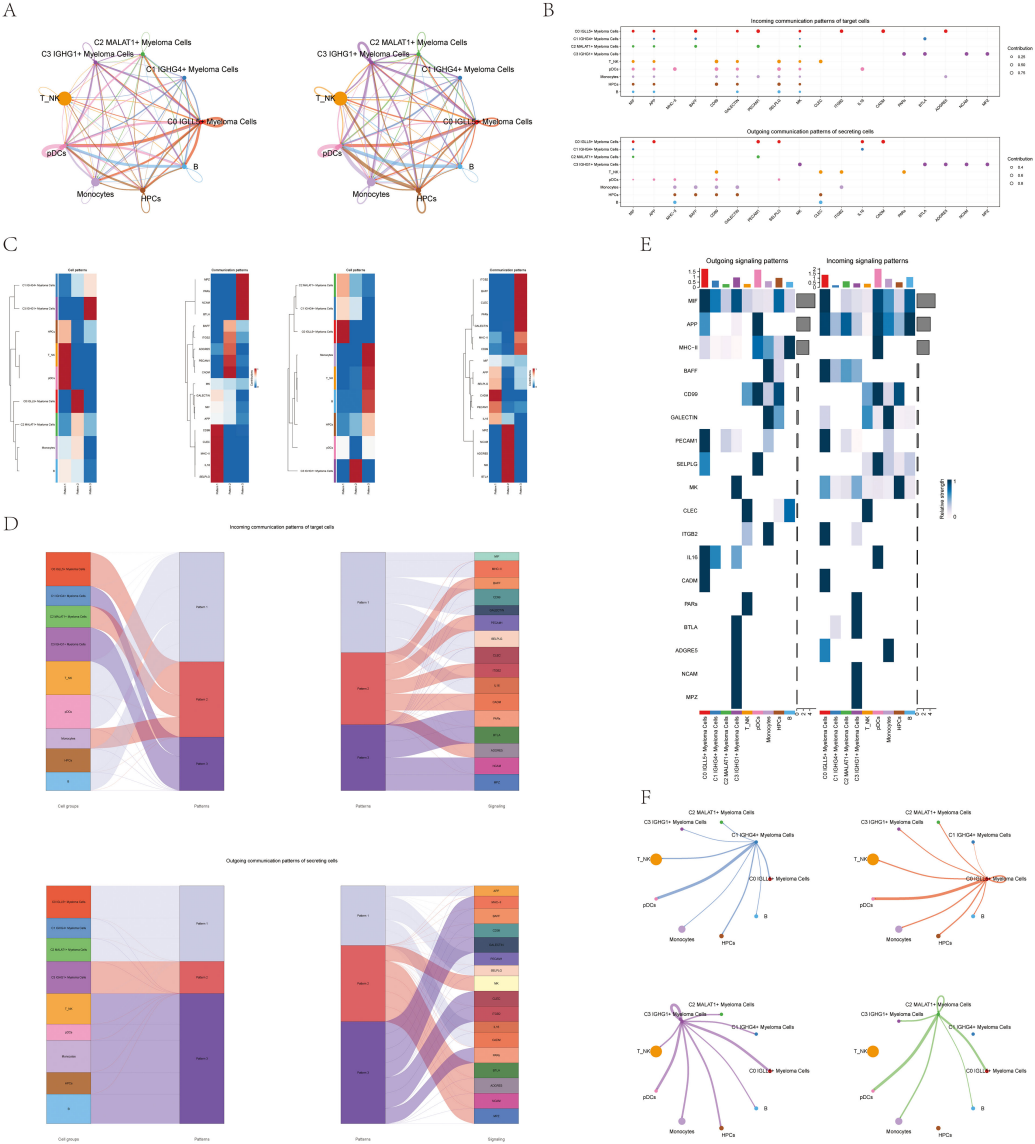
CellChat analysis of interactions between all cells. **(A)** Circular diagrams displayed the quantity of connections (on the left) and the intensity of connections among every cell (on the right). **(B)** The bubble plots for outgoing contributions and incoming contributions displayed the communication patterns among different subpopulations of multiple myeloma cells and other types of cells **(C)** Heatmap illustrated the pattern identification of incoming cells on the left and outgoing cells on the right within all cells. **(D)** Sankey diagrams illustrated the deduced communication patterns of secretory cells, indicating the relationship between these patterns and cell populations, along with signaling pathways. Top: incoming Sankey diagram; Bottom: outgoing Sankey diagram. **(E)** Heatmaps showed the incoming and outgoing signal strength of all cell interactions. **(F)** Interactions between the four cell subpopulations of multiple myeloma cells and other cell types were displayed in chord diagrams.

**Figure 5A** displayed the network visualization, showing connections between cell types as lines with varying thickness to represent the number of interaction pathways. A thicker line indicated a greater number of interactions. Moreover, the thickness of the line symbolized the strength of the connection, where a thicker line denoted a more powerful interaction.

By analyzing this intercellular communication network, we gained insights into the extensive connections and communication pathways between different cell types, providing a systematic understanding of the cellular interactions involved in multiple myeloma and its surrounding microenvironment.

To gain further insights into cellular communication and signaling pathways, we employed the gene expression pattern analysis tools available on CellChat. Our aim was to investigate how different cell types interact and communicate with each other.

Our initial goal was to uncover the connection between hidden communication patterns and secretory cell clusters, in order to understand the patterns of outgoing communication. We discovered three distinct incoming signal patterns: Pattern 1 (involving T_NK cells and pDCs), Pattern 2 (involving C0 IGLL5+ Myeloma Cells), and Pattern 3 (involving C3 IGHG1+ Myeloma Cells). Additionally, we observed three outgoing signal patterns: Pattern 1 (involving C0 IGLL5+ Myeloma Cells), Pattern 2 (involving C3 IGHG1+ Myeloma Cells), and Pattern 3 (involving T_NK cells, B cells, and pDCs). Each pattern was associated with specific incoming and outgoing signals (**Figure 5C**).

We used CellChat to quantitatively measure the ligand-receptor network in order to identify important signals related to the four myeloma cell subgroups. We used pattern recognition techniques to forecast the main incoming and outgoing signals. In the context of multiple myeloma, every cell type has the potential to function as a secretory cell by transmitting signals, as well as a target cell by receiving signals. The interaction between different cell types through ligand-receptor signaling is thought to play a role in the progression of multiple myeloma (**Figure 5B**).

Through examination of the ligand-receptor network and detection of crucial incoming and outgoing signals, we developed a more profound comprehension of the signaling connections among various cell types in multiple myeloma. The information offered valuable understanding of the processes involved in the disease’s development and advancement.

CellChat used a pattern recognition technique relying on non-negative matrix factorization to comprehend the coordination of functions among various cell populations and signaling pathways. Through this analysis, we discovered three outgoing signal patterns and three incoming signal patterns. The output revealed that a significant portion of the incoming signaling in myeloma cells was characterized by pattern 2 and pattern 3. These patterns indicated various routes, such as BAFF, PECAM1, CADM, and more. On the other hand, the signaling from T_NK cells, B cells, pDCs, and HPCs exhibited pattern 1, which was associated with pathways such as MIF, CD99, CLEC, and others.

These findings provided insights into the coordinated communication and signaling between different cell populations in multiple myeloma. Analyzing the worldwide communication trends and important indicators can enhance our comprehension of how different pathways and cell types interact, leading to a more thorough understanding of the illness.

Furthermore, the analysis of target cell communication patterns revealed that the outgoing signaling from myeloma cells was predominantly characterized by pattern 1 and pattern 2. These patterns involved signaling pathways such as APP, MK, CADM, PECAM1, BTLA, and others. In contrast, the signals released by T_NK cells, B cells, pDCs, HPCs, and Monocytes showed a different pattern, pattern 3, influenced by pathways like CLEC and CD99 (**Figure 5D**).

Interestingly, we observed that MIF played a significant role in both incoming and outgoing signaling of C0 IGLL5+ Myeloma cells. Additionally, CLEC was found to be highly expressed in T_NK cells and played a crucial role in both incoming and outgoing patterns (**Figure 5E**).

A chord diagram was used to illustrate the connections between the four subgroups of myeloma cells and other types of cells (**Figure 5F**). The diagram offered a thorough depiction of the interaction and communication among various cell groups in the setting of multiple myeloma.

The results illuminated the complex web of communication and signaling interactions among different cell types in cases of multiple myeloma. The identification of key signaling pathways and their associations with specific cell populations contributed to our understanding of the disease’s complexity and may have implications for developing targeted therapeutic approaches.

### Visual analysis of MIF and CLEC signaling pathways

**Figure 6:**
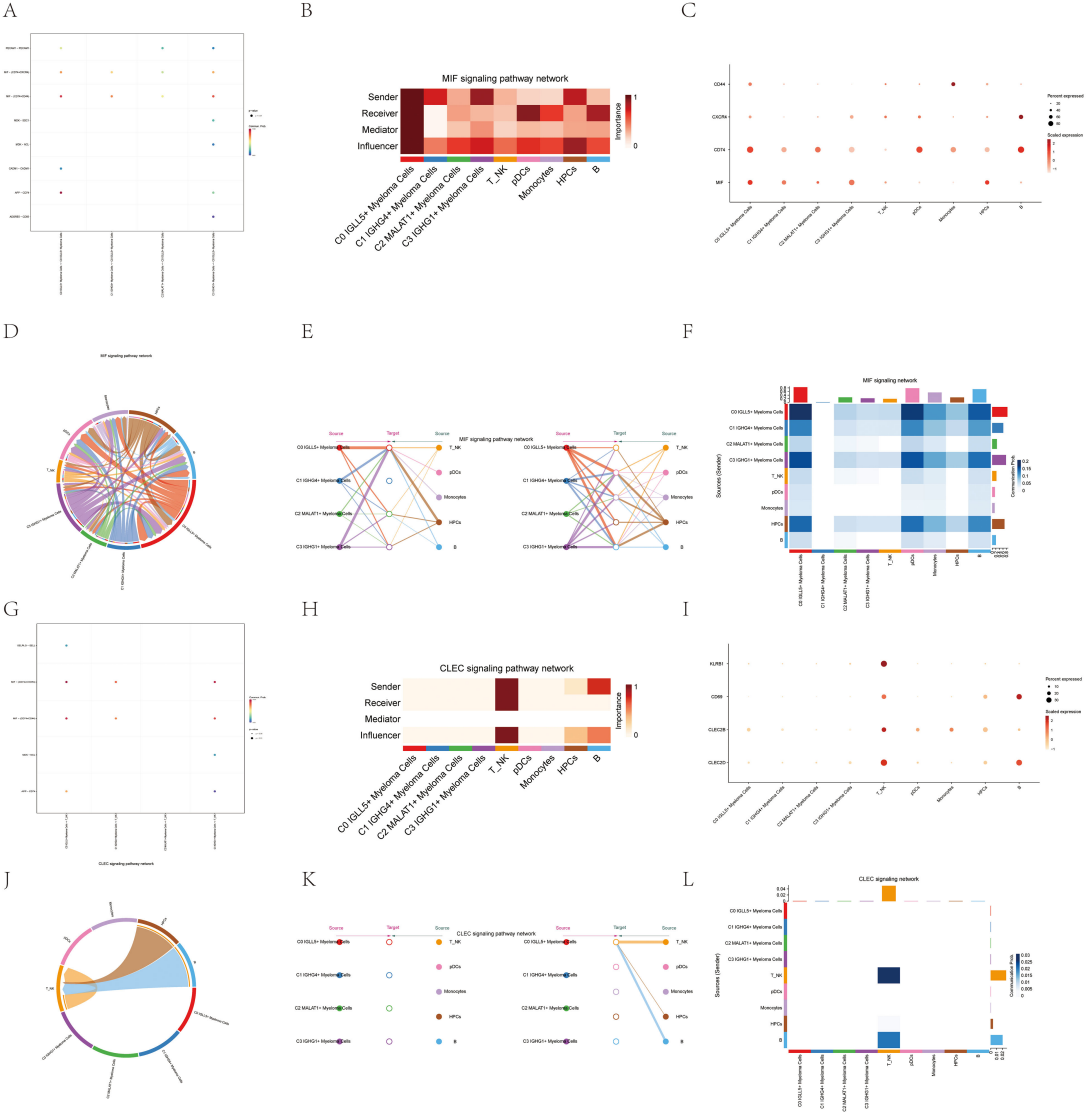
Interactions analysis between cells in the MIF and CLEC signaling pathways. **(A)** A graphical representation displayed the connections between ligands and receptors among the four cell subgroups of multiple myeloma and C0 IGLL5+ Myeloma Cells. **(B)** The centrality scores of the MIF signaling pathway network were visualized on a heatmap, showing the varying significance of each module group. **(C)** Dot plot showed the relationships between all cell types in multiple myeloma and the marker genes. The size of the dots represented the percentage of gene expression in the subpopulations, and the color intensity represented the expression level. **(D)** Circular plot showed the cell interactions in the MIF signaling pathway, with multiple myeloma cells as receivers. **(E)** The hierarchical chart illustrated the connections among multiple myeloma cells and other cells within the MIF signaling pathway. Solid circles and hollow circles represented the source and target cell types, respectively.The color of the outer circles matched the origin of the signals. **(F)** Heatmap displayed the cell interactions in the MIF signaling pathway. **(G)** The dot plot illustrated the connections between the ligand and receptor in the four different cell subpopulations of multiple myeloma and T_NK Cells. **(H)** The centrality scores of the CLEC signaling pathway network were shown on a heatmap, revealing the significance of each module group. **(I)** Dot plot showed the relationships between all cell types in multiple myeloma and the marker genes. The size of the dots represented the percentage of gene expression in the subpopulation, and the color intensity represented the expression level. **(J)** Circular plot showed the cell interactions in the CLEC signaling pathway, with multiple myeloma cells as receivers. **(K)** The hierarchical chart illustrated the connections among multiple myeloma cells and other cells in the CLEC signaling pathway. Solid circles and hollow circles represented the source and target cell types, respectively. The color of the outer circles matched the origin of the signals. **(L)** Heatmap displayed the cell interactions in the CLEC signaling pathway.

To investigate the functional pathways of MIF and CLEC signaling, we conducted a visual analysis of these pathways. Studies of the role of MIF (which largely functions via the type II transmembrane receptor CD74) in prostate, bladder and kidney cancers suggested that it is a pro-tumorigenic factor in genitourinary malignancy (59). Some previous studies have mentioned that there is a correlation between CLEC and cancer (60). First, we examined the ligand-receptor relationship between the C0 subgroup and other subgroups in the MIF signaling pathway using a dot plot (**Figure 6A**). This plot demonstrated the interactions between C0 and other subgroups within the MIF pathway.

Through the use of algorithmic calculations, we assessed the significance of individual cell types and categorized them as facilitators and drivers of MIF signaling in cell communication. This analysis, known as “centrality measurement,” provided insights into the key players in the MIF signaling pathway. The myeloma cell subgroup C0 IGLL5+ displayed the highest level of expression in the MIF signaling pathway, as depicted in **Figure 6B**.

Additionally, a scatter plot was created to display the gene expression related to the MIF pathway in different cell types. Notably, the myeloma cell subgroup C0 IGLL5+ Myeloma Cells exhibited high expression of genes related to the MIF pathway (**Figure 6C**).

The importance of the MIF signaling pathway was underscored by these results, especially within the C0 subgroup of myeloma cells. The dot plot analysis provided a comprehensive view of gene expression within different cell types, emphasizing the role of the C0 subgroup in the MIF pathway.

Through clarifying the roles of MIF and CLEC signaling pathways and their connections to distinct cell groups, we enhanced our comprehension of the molecular processes involved in multiple myeloma. This information had the potential to aid in the creation of specific treatments and actions designed to understand these communication pathways for medical purposes.

**Figure 6D** displayed the ligand-receptor interactions between myeloma cells and other cell types in a visual chord diagram. To further analyze the MIF signaling pathway, we categorized all nine identified cell types in myeloma as potential MIF source cells. On the left side of **Figure 6E**, we selected four cell types as potential target cells. The stratification plot revealed that only C0 IGLL5+ Myeloma Cells had the ability to target the MIF released by all nine cell types.

Conversely, when the remaining five cell types mentioned in **Figure 6E** were examined as potential recipients, the graph showed the attraction of MIFs produced by all nine cell types, as shown in **Figure 6E**.

To provide more detailed insights into the cell-cell interactions within the MIF signaling pathway, **Figure 6F** displayed the specifics of these interactions.

These analyses highlighted the complex and multifaceted nature of the MIF signaling pathway in multiple myeloma. The stratification plots demonstrated the unique targeting capabilities of C0 IGLL5+ Myeloma Cells and emphasized their role in the MIF-mediated communication network. Understanding the specific interactions between different cell types in the MIF signaling pathway can aid in the development of targeted therapies that disrupt these interactions and potentially attenuate disease progression.

To investigate the involvement of T_NK cells in the CLEC signaling pathway, we visualized the pathway and analyzed their interactions with other subgroups. The dot plot (**Figure 6G**) demonstrated the ligand-receptor relationships between T_NK cells and other subgroups within the CLEC signaling pathway.

By employing the “centrality measurement” approach, we determined the relative expression levels of different cell types in the CLEC signaling pathway. As shown in **Figure 6H**, T_NK cells exhibited the highest expression among all cell types in the CLEC pathway, indicating their significant involvement in this signaling cascade.

**Figure 6I**, the dot plot, provided additional visualization of gene expression levels related to the CLEC pathway in various cell types. Notably, T_NK cells displayed high expression of genes relevant to the CLEC signaling pathway.

To visualize the ligand-receptor interactions specifically involving T_NK cells, the chord plot (**Figure 6J**) was utilized. It provided a comprehensive view of the ligands between T_NK cells and other cells within the CLEC signaling pathway.

The stratified plots (**Figure 6K**) demonstrated the expression patterns of T_NK cells in response to the ligands released by other cell types within the CLEC pathway.

To delve deeper into the specific cell-cell interactions within the CLEC signaling pathway, **Figure 6L** showcased the details of these interactions.

These analyses shed light on the role of T_NK cells in the CLEC signaling pathway of multiple myeloma. The various types of plots, including dot plots, chord plots, and stratified plots, along with detailed information on cell-cell interactions, offered important insights into the expression patterns and relationships of T_NK cells. Comprehending the intricacies of these interactions may enhance our comprehension of the CLEC signaling pathway and its potential as a target for treatment in cases of multiple myeloma.

### Expression of stemness genes in myeloma cell subgroups

The CytoTrace analysis revealed that C0 IGLL5+ Myeloma Cells exhibited the highest stemness among the different myeloma cell subgroups (**Figure 3B**). In order to delve deeper into the stemness of these subcategories, an analysis was conducted on the expression of genes associated with stemness.

**Figure 7:**
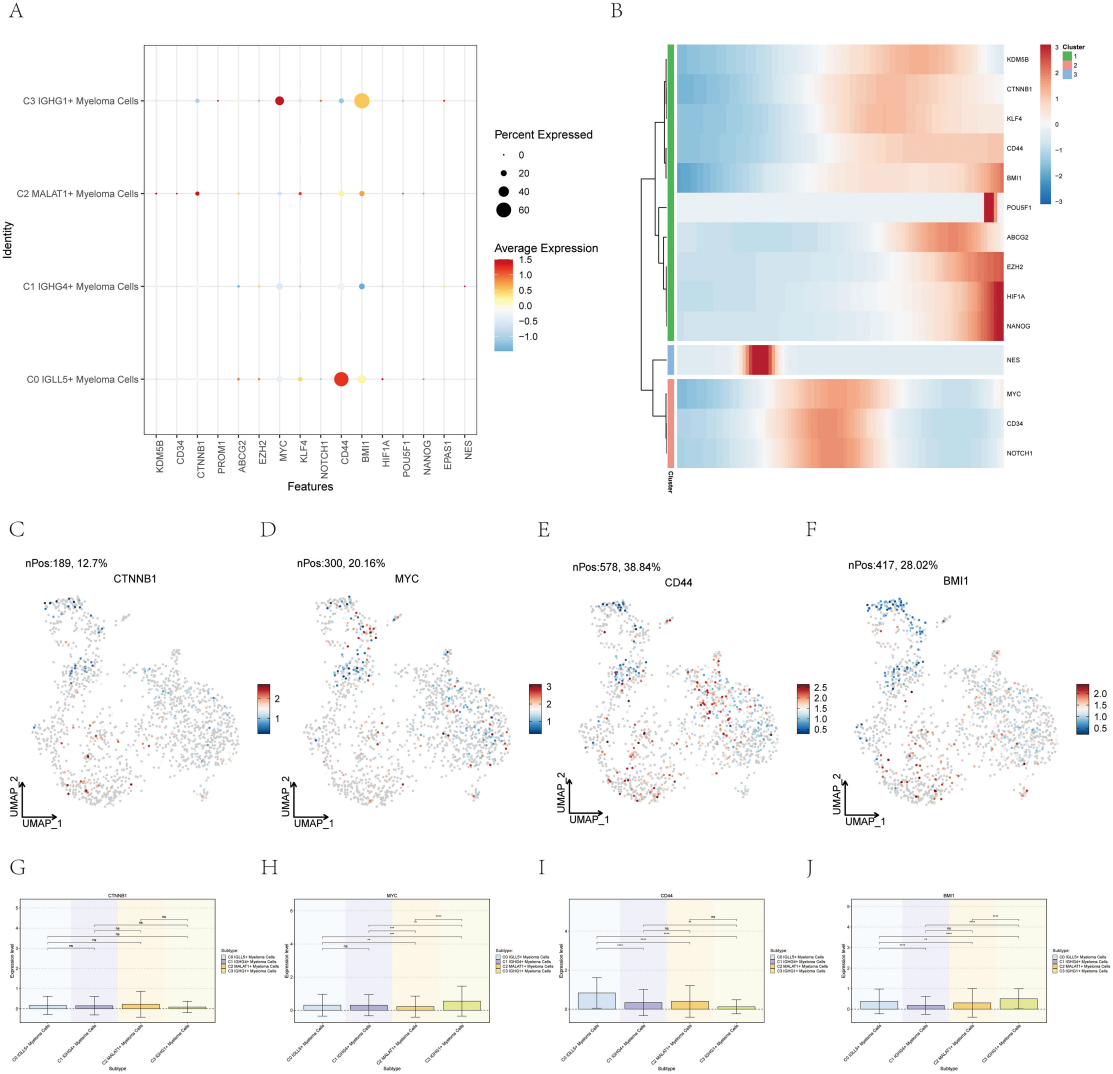
Expression of stemness-related genes in multiple myeloma cell subpopulations. **(A)** A graph displayed the levels of stem cell-related genes in the four distinct groups. **(B)** The heatmap showed the levels of expression for genes related to stemness. **(C–J)** UMAP and box plots demonstrated the variation in gene expression related to stemness in different subgroups of multiple myeloma cells. *, p ≤ 0.05; **, p<0.01; ***, p<0.001; ****, p<0.0001 indicated significant differences; ns indicated no significant difference.

The dot plot (**Figure 7A**) visualized the expression levels of stemness-related genes across the myeloma cell subgroups, providing insights into their relative expression patterns.

A heatmap (**Figure 7B**) was created to fully comprehend the gene expression related to stemness in myeloma cells. This heatmap showcased the expression profiles of stemness-related genes across the different myeloma cell subgroups, allowing for a comparative analysis.

Furthermore, box plots (**Figures 7C–J**) were utilized to emphasize the varying expression of genes associated with stemness within the different subgroups of myeloma cells. These plots provided statistical summaries of the gene expression levels, enabling a quantitative comparison between the subgroups.

By employing these visualization techniques, we gained insights into the expression profiles and differential expression patterns of stemness-related genes across the myeloma cell subgroups. This information contributed to our understanding of the stemness characteristics and heterogeneity within multiple myeloma, which can have implications for disease progression and potential therapeutic strategies.

### Analysis of gene regulatory networks of myeloma cell subgroups

In order to identify the core transcription factors (TFs) active in the different myeloma cell subgroups, a SCENIC analysis was conducted. Gene regulatory networks were inferred for each myeloma cell subgroup using the pySCENIC tool.

**Figure 8:**
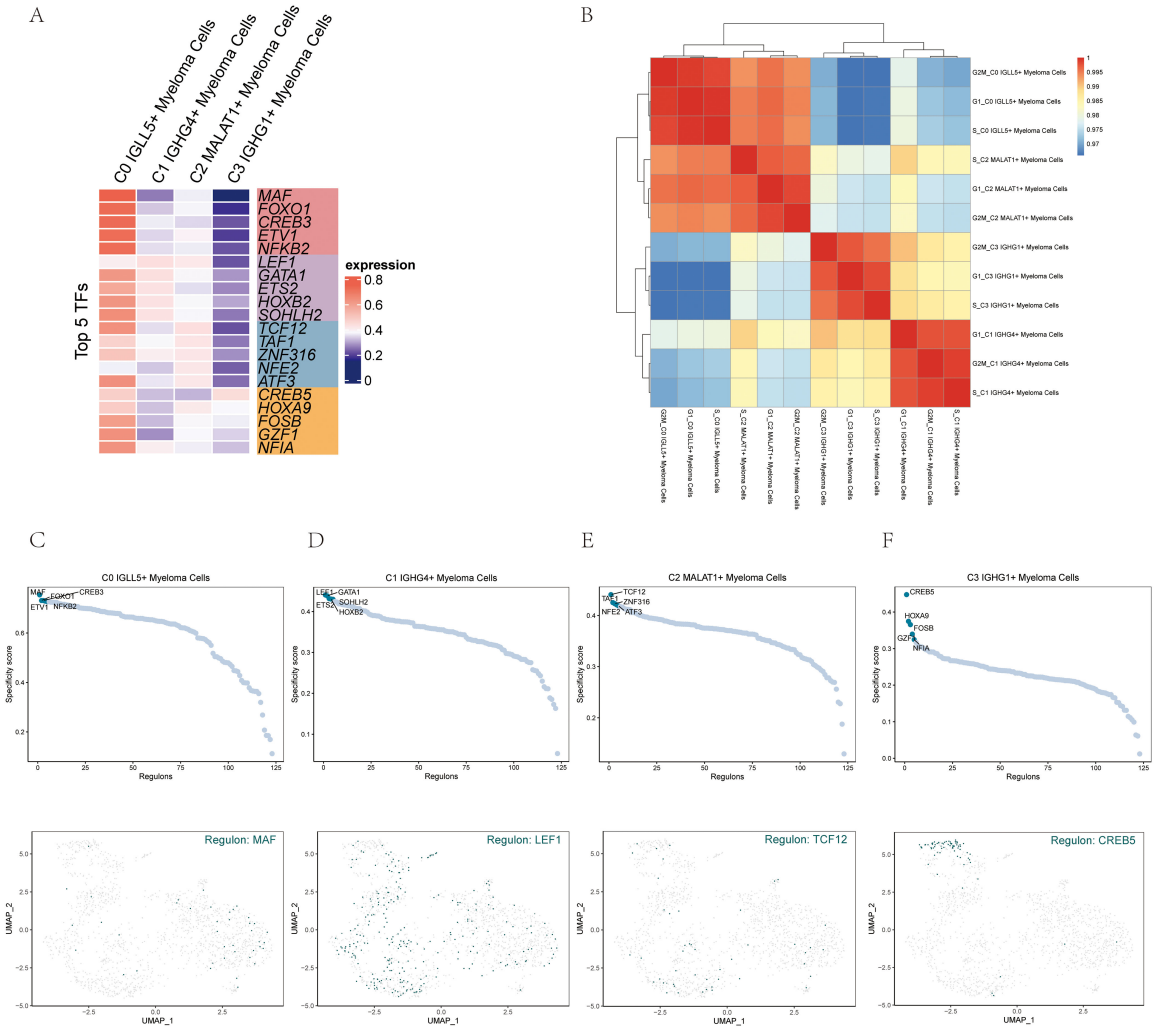
Gene regulatory network analysis of multiple myeloma cell subpopulations. **(A)** A visualization using colors represented the activity of the top 5 transcription factors (TFs) in the various cell subgroups of multiple myeloma. **(B)** The heatmap displayed the relationships among the four cell subgroups of multiple myeloma and stages. **(C–F)** The ranking of regulators was displayed based on the regulatory subnetwork-specific scores (RSS) in the four cell subpopulations of multiple myeloma (top) were shown. The UMAP plots of each subpopulation highlighted the top-ranked genes (bottom).

Based on previous studies that specifically modulated the activity of these cell types, the most activated TFs in each myeloma cell subgroup were determined. Specifically, the activated TFs included MAF in C0 IGLL5+ Myeloma Cells, LEF1 in C1 IGHG4+ Myeloma Cells, TCF12 in C2 MALAT1+ Myeloma Cells, and CREB5 in C3 IGHG1+ Myeloma Cells (**Figure 8A**). It was probable that these transcription factors will have important functions in controlling the expression of genes and cellular activities in their specific groups.

The heatmap (**Figure 8B**) visualized the relationship between the four myeloma cell subgroups and different phases, providing insights into their gene expression patterns and potential regulatory mechanisms.

To provide a more visual representation of gene expression, scatter plots and UMAP plots were employed (**Figures 8C–F**). These plots allowed for the visualization of gene expression patterns within the myeloma cell subgroups and highlighted the distinct clusters or subpopulations present.

To rank the regulators in the myeloma cell subgroups based on their regulon specificity score (RSS), the UMAP plots were utilized. This ranking provided information on the importance and specificity of different regulators within each myeloma cell subgroup.

By leveraging these analyses and visualization techniques, we gained a deeper understanding of the core TFs active in each myeloma cell subgroup and their potential regulatory networks. The scatter plots, UMAP plots, and regulon specificity scores contributed to our knowledge of gene expression patterns and regulatory mechanisms within multiple myeloma, offering insights into the underlying biology and potential therapeutic targets.

### Identification of TF regulatory submodules in myeloma cell subgroups

**Figure 9:**
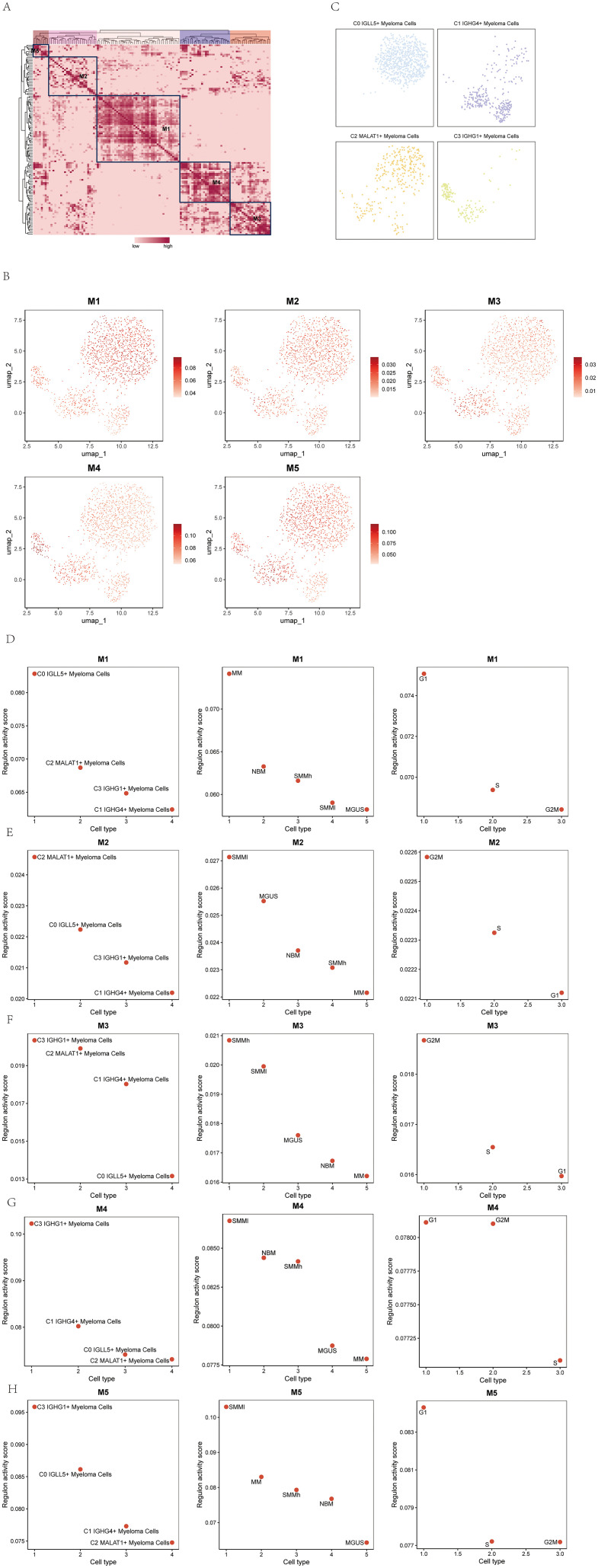
Identification of TF regulatory module in multiple myeloma cell subpopulations. **(A)** Identification of five regulatory modules in multiple myeloma cell subpopulations based on the connectivity specificity index (CSI) matrix were shown. **(B)** UMAP plots showed the expression of the five TF regulatory modules in multiple myeloma cell subpopulations. **(C)** Scatter plots illustrated the distribution of multiple myeloma cell subpopulations after SCENIC analysis. **(D-H)** Scatter plots showed the transcriptional activity scores of the four cell subpopulations of multiple myeloma on modules M1-M5 (left); scatter plots showed the transcriptional activity scores of the five groups of multiple myeloma cells on modules M1-M5 (middle); scatter plots showed the transcriptional activity scores of the three phases of multiple myeloma cells on modules M1-M5 (right).

In the study, we further identified five regulatory modules of the myeloma cell subgroups using the connection specificity index (CSI) matrix. These modules were labeled as M1, M2, M3, M4, and M5 (**Figure 9A**). Each module represented a distinct set of regulatory interactions within the myeloma cell subgroups.

UMAP plots were created to illustrate the expression patterns of the five TF regulatory modules in the myeloma cell subgroups (**Figure 9B**). The graphs visually depicted the levels of expression and distribution of regulatory modules among various subgroups of myeloma cells.

Scatter plots were employed to demonstrate the distribution of myeloma cell subgroups after the SCENIC analysis (**Figure 9C**). These plots allowed for the visualization of the clustering and relationships between the subgroups based on their transcriptional profiles.

To assess the transcriptional activity scores of the myeloma cell subgroups on the M1-M5 modules, scatter plots were utilized. The left panel of **Figures 9D–H** displayed the transcriptional activity scores of the four cell subgroups of myeloma cells on the M1-M5 modules. The middle panel showed the transcriptional activity scores of the five myeloma cell subgroups on the M1-M5 modules. Finally, the right panel presented the transcriptional activity scores of the three phases of myeloma cells on the M1-M5 modules. These scatter plots provided insights into the transcriptional activities and regulatory dynamics within the myeloma cell subgroups and their respective phases.

Through the use of these visualization methods, we acquired a thorough comprehension of the regulatory modules and their patterns of expression in the myeloma cell subcategories. The scatter plots facilitated the comparison of transcriptional activity scores between the subgroups and phases, shedding light on the regulatory mechanisms underlying the development and progression of multiple myeloma.

### Experimental validation

**Figure 10:**
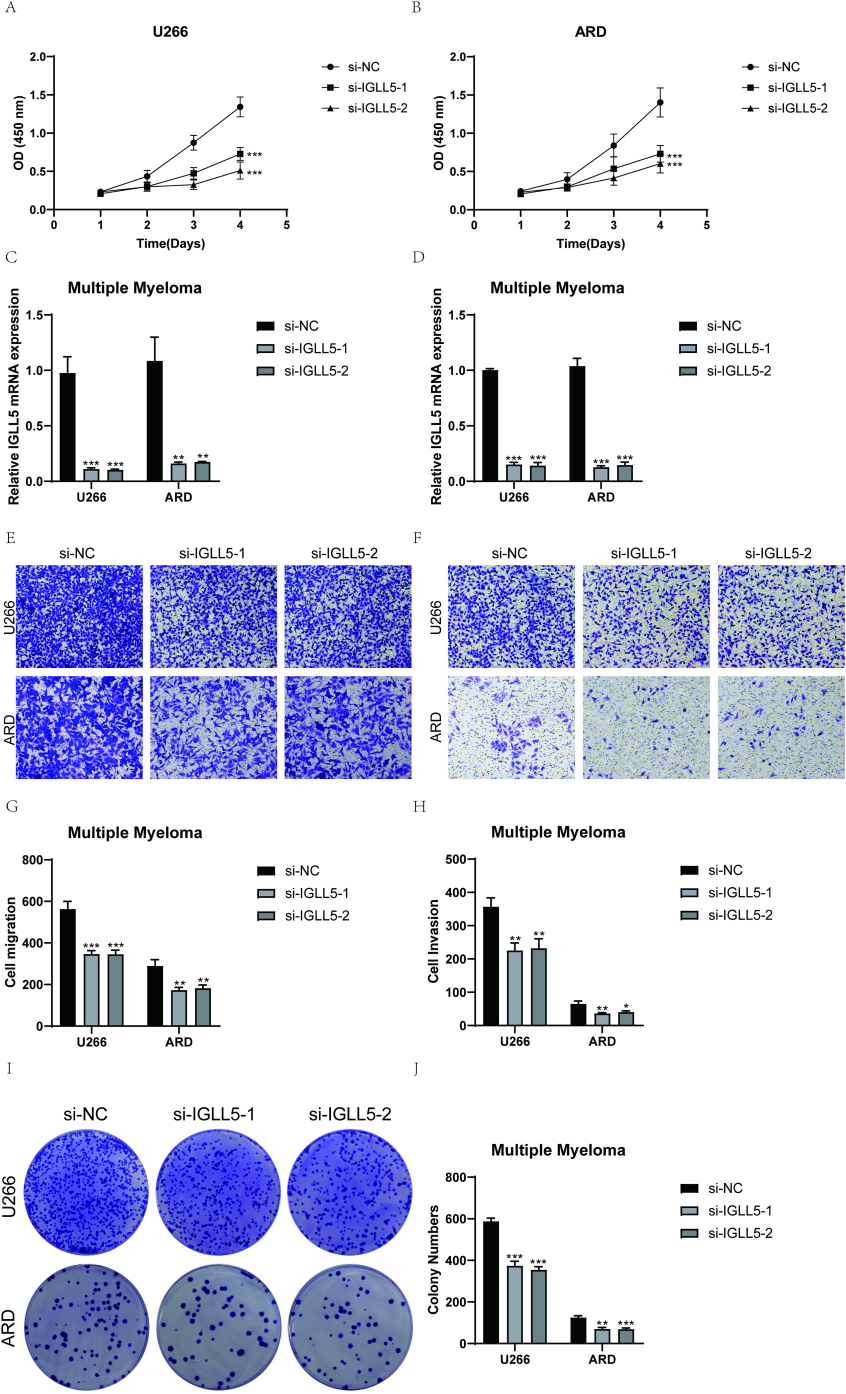
IGLL5 significantly affected the proliferation and migration of multiple myeloma cell lines. **(A)** Following IGLL5 knockdown, a notable reduction in cell viability of U266 cell line was observed using a CCK-8 assay. **(B)** CCK-8 test indicated a notable reduction in the survival of ARD cells following the suppression of IGLL5. **(C, D)** Bar graph showed the relative IGLL5 mRNA expression in U266 and ARD cell lines. **(E, F)** Transwell assay showed a significant decrease in cell migration of both U266 and ARD cell lines after IGLL5 knockdown. **(G, H)** Cell invasion of both U266 and ARD cell lines significantly decreased after IGLL5 knockdown. **(I, J)** Plate cloning experiment showed a significantly lower cell colony formation in the IGLL5 knockdown group compared to the negative control group. *, p ≤ 0.05; **, p<0.01; ***, p<0.001 indicated significant differences; ns indicated no significant difference.

U266 and ARD cell lines were selected for *in vitro* functional experiments to validate the function of IGLL5. Controls were established with a negative control group and an infection group with IGLL5 knockdown. Following IGLL5 knockdown, the cell activity assay revealed a notable reduction in the proliferation capacity of U266 and ARD cells, demonstrating statistically significant variances (**Figures 10A–D**).

IGLL5 knockdown significantly attenuated the migration and invasion abilities of U266 and ARD cells. The transwell assay showed a notable decrease in the stained area of both cell lines with suppressed IGLL5 compared to the negative control group (**Figures 10E–H**).

In the plate cloning experiment, the colony forming efficiency was analyzed and calculated, and the results showed that IGLL5 knockdown significantly reduced the colony formation of U266 and ARD cell lines (**Figures 10I, J**).

**Figure 11:**
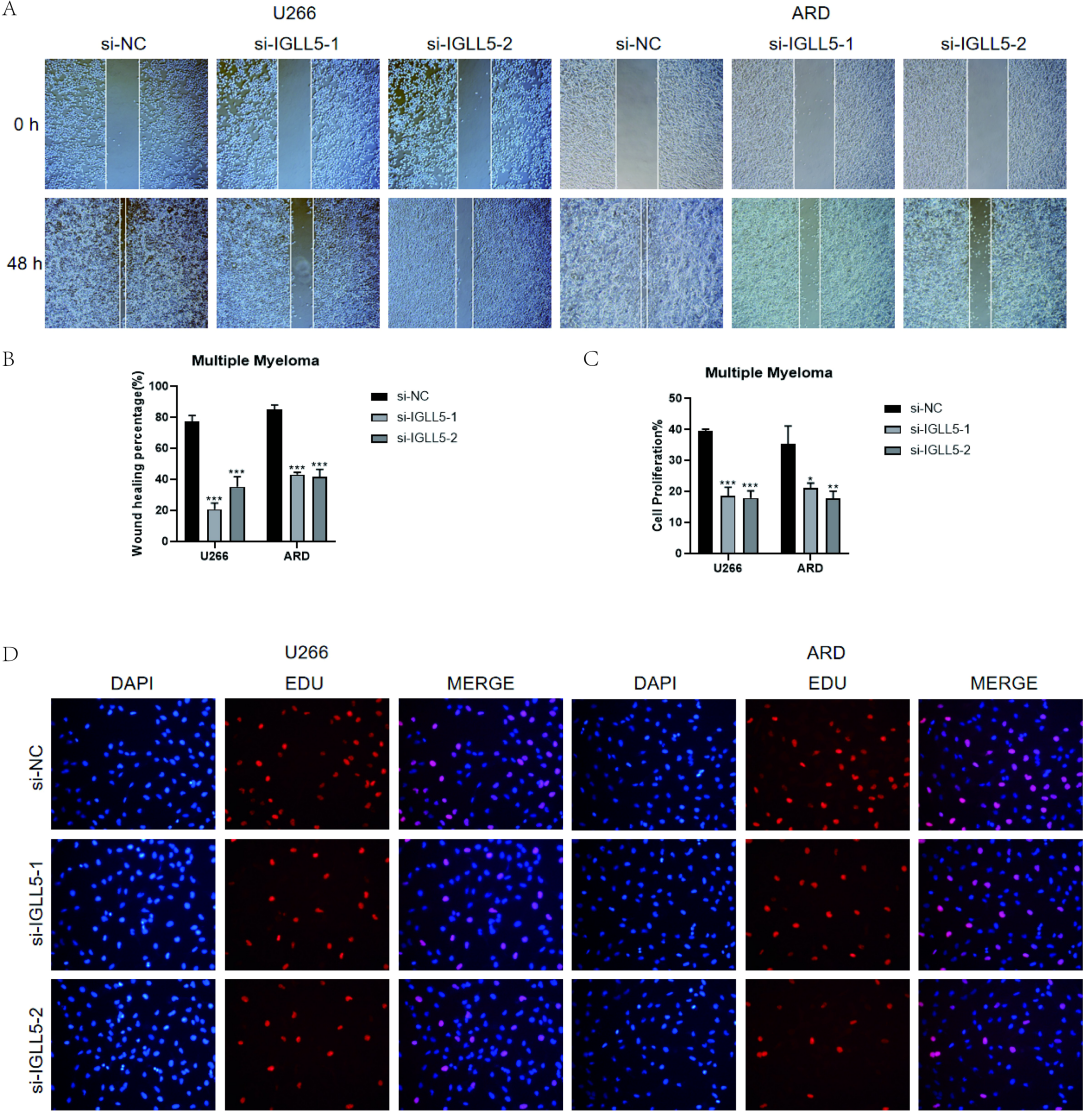
*In vitro* validation of IGLL5. **(A, B)** Scratch assay demonstrated a notable widening of the scratch after 48 hours in U266 and ARD cell lines post IGLL5 knockdown, in comparison to the negative control group. *, p ≤ 0.05; **, p<0.01; ***, p<0.001 indicated significant differences; ns indicated no significant difference. **(C, D)** EdU staining showed a decrease in cell proliferation ability after IGLL5 knockdown.

Furthermore, the results of the wound-healing assay demonstrated that IGLL5 knockdown significantly impaired the migration ability of U266 and ARD cell lines compared to the negative control group (**Figures 11A, B**).

EdU staining indicated a notable decrease in the cell count of U266 and ARD cell lines with IGLL5 knockdown compared to the negative control group (**Figures 11C, D**).

Therefore, through multiple experiments, it was found that IGLL5 knockdown can reduce the migration, invasion, and proliferation abilities of multiple myeloma cells.

### Expression of myeloma cells subsets on the MMP and TIMP pathways

**Figure 12:**
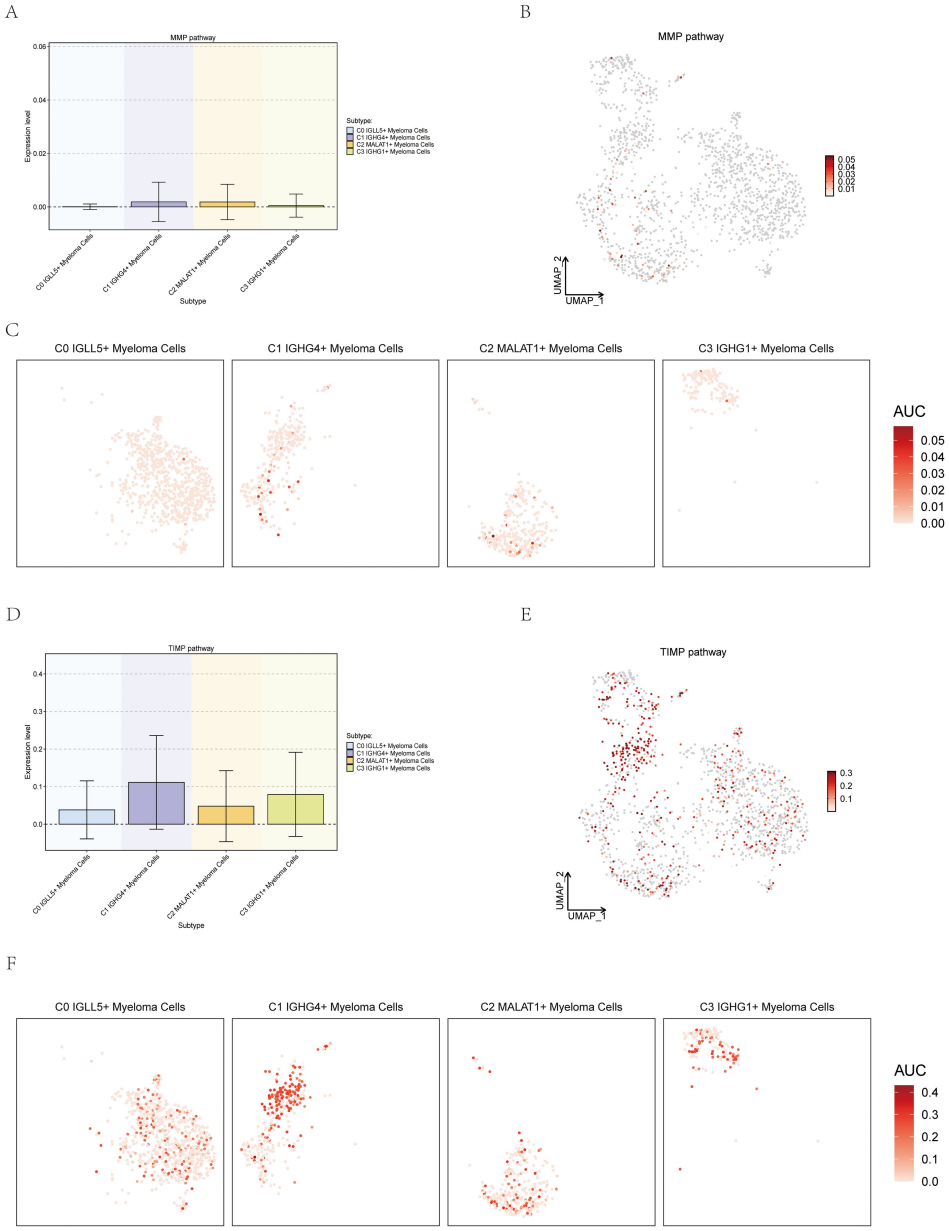
Expression of myeloma cells subsets on the MMP and TIMP Pathways. **(A–C)** Box plot, UMAP plot, and facet plots showed the expression of myeloma cells subsets on the MMP pathways. **(D–F)** Box plot, UMAP plot, and facet plots showed the expression of myeloma cells subsets on the TIMP pathways.

To investigate the potential correlation between myeloma cells and metal ions, the study showcased the expression of different subsets of Myeloma Cells on the pathways which were related with metal ions. Matrix metalloproteinases (MMPs) are members of the zinc-dependent endopeptidase family and are capable of degrading almost all protein components in the extracellular matrix (ECM) (61). Members of the tissue inhibitor (TIMP) family of metalloproteinases are highly regarded as natural inhibitors of cancer-promoting metalloproteinases (62). The association between MMP and TIMP and a variety of cancers had been previously documented, so we tried to explore the association between them and MM. Four subsets were visualized through box plot, UMAP plot, and facet plots, revealing that C0 IGLL5+ Myeloma Cells’ expression on the MMP and TIMP pathways (**Figures 12A–F**).

## Discussion

In this investigation, the utilization of scRNA-Seq technology enabled the comprehensive characterization of tumor heterogeneity and the tumor immune microenvironment in multiple myeloma (MM). Every unique cell category found in MM, such as Monocytes, Hematopoietic Progenitor Cells (HPCs), Plasmacytoid Dendritic Cells (pDCs), B cells, T_NK cells, and Plasma cells, were successfully identified. Previous research findings had established multiple myeloma as a malignant neoplasm originating from the blood system, distinguished by the presence of aberrant clonal plasma cells within the bone marrow (57).

To further investigate the malignant plasma cells, here referred to as myeloma cells, dimensionality reduction techniques were employed to cluster these cells. This analysis resulted in the identification of four distinct cell subsets: C0 IGLL5+ Myeloma Cells, C1 IGHG4+ Myeloma Cells, C2 MALAT1+ Myeloma Cells, and C3 IGHG1+ Myeloma Cells. Thorough examination of these cell subgroups from different angles resulted in a complete understanding of their molecular and cellular characteristics.

By employing slingshot, monocle, and CytoTRACE, we successfully demonstrated the differentiation of myeloma cells along a pseudotime trajectory. Through these analyses, we identified the specific subgroup of myeloma cells that became the focus of our study, namely the C0 IGLL5+ Myeloma Cells subgroup. The selection of this subgroup was based on several key factors.

Firstly, in the predicted ordering generated by CytoTRACE, the expression level of C0 IGLL5+ Myeloma Cells exhibited the highest magnitude, indicating their superior differentiation ability compared to other subgroups. This finding suggested that the cells within this subgroup possess the highest level of cell stemness (63).

Secondly, in the pseudotime series analysis, a significant proportion of cells within the C0 IGLL5+ Myeloma Cells subgroup were located at the terminal stage of the differentiation trajectory. This observation indicated a high degree of malignancy associated with this subgroup. It is worth noting that cell stemness is closely intertwined with tumor metastasis (64), further supporting the notion that the high stemness of this subgroup is closely linked to its malignancy.

Lastly, the analysis conducted using slingshot revealed that the C0 IGLL5+ Myeloma Cells subgroup was situated at the endpoint of lineage1, which corresponded to the conclusion drawn from the aforementioned analyses. This alignment further validated our perspective on the differentiation and malignancy of this subgroup.

In summary, the selection of the C0 IGLL5+ Myeloma Cells subgroup was based on their robust differentiation ability, their positioning at the end of the differentiation trajectory with a high degree of malignancy, and their alignment with lineage1 as indicated by slingshot analysis. These findings collectively supported the notion that this subgroup held significant relevance to the progression of multiple myeloma.

Prior research had offered important information on the importance of the gene known as IGLL5 in relation to multiple myeloma. These studies had consistently demonstrated that the expression level of IGLL5 is significantly elevated under disease conditions compared to the precursor state (65). Furthermore, alterations in IGLL5 had been linked to a higher likelihood of disease advancement (66). Notably, an independent analysis revealed that IGLL5 mutation serves as a contributing factor to the heightened risk of early progressive disease in multiple myeloma patients (67).

These findings led us to theorize that the C0 IGLL5+ Myeloma Cells subgroup is strongly linked to the advancement of multiple myeloma. The elevated expression level of IGLL5 and its identified mutations in disease conditions strongly suggested its involvement in disease development and progression. Previous studies have found that IGLL5, a other biomarker that is more specifically expressed under dominant disease conditions, has the potential to serve as an emerging biomarker for targeted therapy (65).Therefore, this subgroup, characterized by the expression of IGLL5, held significant relevance in understanding the mechanisms underlying the progression of multiple myeloma.

By utilizing the CellChat communication pattern analysis, we were able to uncover the coordinated interactions between the C0 IGLL5+ Myeloma Cells subgroup and other cell types. The CellChat analysis offered important information on the connections among different types of cells in myeloma. By employing the CellChat communication pattern analysis, we delved deeper into the complex web of cellular interactions within the context of multiple myeloma. This innovative approach allowed us to unravel the intricate relationships between different cell types, shedding light on the dynamic interplay within the tumor microenvironment.

In particular, our study shed new insights into the coordinated interactions involving the C0 IGLL5+ Myeloma Cells subgroup and various other cell types. We discovered a network of communication pathways through which these cells engage with their surrounding microenvironment. This intricate intercellular crosstalk is crucial for the development and progression of multiple myeloma. Through this analysis, we identified specific communication patterns and their corresponding signal pathway expressions. Notably, the MIF (Macrophage Migration Inhibitory Factor) signal pathway was found to be associated with the C0 IGLL5+ Myeloma Cells subgroup, indicating its importance in the signaling network of this subgroup. The MIF signaling pathway was probably important in the biological processes and functions of the C0 IGLL5+ Myeloma Cells subgroup.

Additionally, the analysis revealed the CLEC signal pathway, which was associated with T_NK cells. This finding suggested that the CLEC signal pathway is an important signaling mechanism in the interaction between T_NK cells and the C0 IGLL5+ Myeloma Cells subgroup. Exploring the distinct signal pathways linked to various cell types can offer valuable understanding of the mechanisms behind cell-cell interactions and their potential impact on multiple myeloma.

In summary, the CellChat communication pattern analysis identified the MIF signal pathway as an important signaling mechanism associated with the C0 IGLL5+ Myeloma Cells subgroup and the CLEC signal pathway as a significant pathway corresponding to T_NK cells. The results illuminated the synchronized responses and connections among the C0 IGLL5+ Myeloma Cells subgroup and various cell types, offering important insights into the tumor microenvironment and possible treatment targets for multiple myeloma.

The findings from the analysis of the MIF signaling pathway further supported the strong correlation between the C0 IGLL5+ Myeloma Cells subgroup and this pathway. The results indicated that the C0 IGLL5+ Myeloma Cells subgroup had the largest number and the highest centrality score within the MIF signaling pathway. This implication indicated that this particular subset was essential in initiating and controlling this process. Moreover, previous studies had reported that MM cells express high levels of MIF (68), which aligned with the current findings and reinforces the significance of the C0 IGLL5+ Myeloma Cells subgroup in this study. These results collectively highlighted the importance of this subgroup and its association with the MIF signaling pathway in multiple myeloma.

Regarding the CLEC signaling pathway, the analysis demonstrated its strong association with T_NK cells. This finding supported the notion that the CLEC signaling pathway is primarily related to the activity and function of T_NK cells. Furthermore, previous studies had indicated that CLEC represented a therapeutic target for immune regulation and was associated with immune responses (69). This suggested that the CLEC signaling pathway may have implications in modulating immune responses and potentially influencing the immune microenvironment in multiple myeloma.

Taken together, the analysis of the MIF and CLEC signaling pathways reinforced the importance of the C0 IGLL5+ Myeloma Cells subgroup and its association with the MIF pathway, as well as the relevance of the CLEC pathway to T_NK cells. The discoveries offered important understanding of the fundamental processes of cell communication and immune control in multiple myeloma, which could aid in creating specific treatments and immune-modifying approaches for the condition.

The SCENIC analysis and gene regulatory network analysis conducted in the study aimed to identify key transcription factors (TFs) in different myeloma cell subgroups and explore the gene regulatory networks associated with these subgroups. The analysis identified five main modules (M1, M2, M3, M4, and M5) within the myeloma cell subgroups. Additionally, the most activated TFs in each subgroup within the M1-M5 modules were identified as follows: MAF in the C0 IGLL5+ Myeloma Cells subgroup, LEF1 in the C1 IGHG4+ Myeloma Cells subgroup, TCF12 in the C2 MALAT1+ Myeloma Cells subgroup, and CREB5 in the C3 IGHG1+ Myeloma Cells subgroup.

MMP (matrix metalloproteinases) affects tissue integrity and promotes cancer cell invasion and metastasis. MMP is one of the underlying causes of multiple myeloma bone disease. TIMP (tissue inhibitors of metalloproteinases), as an important regulatory factor for MMP hydrolysis or activation, also participates in the development and progression of multiple myeloma and the formation of bone disease. Understanding the intricate relationship between MMP, TIMP, and multiple myeloma is crucial for identifying potential therapeutic targets and developing strategies to mitigate the devastating consequences of bone disease. By elucidating the specific mechanisms underlying the dysregulation of MMP and TIMP, researchers can pave the way for novel treatment approaches aimed at restoring the balance of these crucial factors and ultimately improving the outcomes for multiple myeloma patients.

The examination yielded important information on the regulatory systems and crucial transcription factors linked to various myeloma cell subcategories, revealing the molecular processes driving the advancement and spread of multiple myeloma. Identifying the distinct TFs and their regulatory functions within each subgroup could lead to the discovery of therapeutic targets and the creation of individualized treatment plans for individuals with multiple myeloma.

It is crucial to mention that this research had constraints, specifically the limited number of samples chosen, which only reflected a small portion of individuals with multiple myeloma. Hence, additional research with increased sample sizes is necessary to confirm and build upon these results. The researchers recognized this constraint and emphasized the importance of further studies to confirm the involvement of IGLL5+ Myeloma Cells in multiple myeloma. ScRNA-seq could be used to characterize the abundance and functional status of MM-related cell types, as well as to detect cell-to-cell communication, and analyze the relationship between different cell interactions to study the pathophysiological characteristics of the MM microenvironment, thereby predicting the development and prognosis of MM. With the progress of the times, more and more new drugs and materials are used in cancer research (70), the main objective is to enhance the existing knowledge of clinical therapy for multiple myeloma, which could impact treatment choices and the ongoing monitoring of patients in the long run (70–72).

## Conclusion

In this study, scRNA-seq technology was utilized to comprehensively characterize tumor heterogeneity and the tumor immune microenvironment in MM. Distinct cell types present in MM were successfully identified, and four distinct cell subsets of myeloma cells were identified using dimensionality reduction techniques. The C0 IGLL5+ Myeloma Cells subgroup, characterized by the expression of IGLL5, was selected for further investigation due to its robust differentiation ability, high degree of malignancy, and alignment with lineage1. Previous researches had shown a strong connection between this particular subset and the advancement of multiple myeloma, underscoring the importance of IGLL5 in the progression of the disease. CellChat analysis revealed coordinated interactions between the C0 IGLL5+ Myeloma Cells subgroup and other cell types, with the MIF and CLEC signaling pathways identified as important pathways associated with this subgroup. The analysis of the MIF and CLEC signaling pathways reinforced the relevance of the C0 IGLL5+ Myeloma Cells subgroup and provided insights into cell-cell interactions and immune regulation in multiple myeloma. Through SCENIC and analysis of gene regulatory networks, important transcription factors and regulatory networks linked to various myeloma cell subgroups were identified, providing valuable understanding of the molecular processes driving disease progression. Nevertheless, the research was constrained, necessitating additional studies with more participants to confirm and build upon these results, aiming to impact the clinical care and monitoring of patients with multiple myeloma.

## Data Availability

The original contributions presented in the study are included in the article/**Supplementary Material**. Further inquiries can be directed to the corresponding authors.

## Supplementary material

Supplementary Figure 1 InferCNV explored single-cell RNA-seq data from myeloma cells to distinguish the cells that we want to study.
