# Supplementary figures and images for: Unveiling the cellular landscape: insights from single-cell RNA sequencing in multiple myeloma
Source: Front Immunol. 2024 Aug 30;15:1458638. doi: 10.3389/fimmu.2024.1458638 (PMC11392786; doi:10.3389/fimmu.2024.1458638)

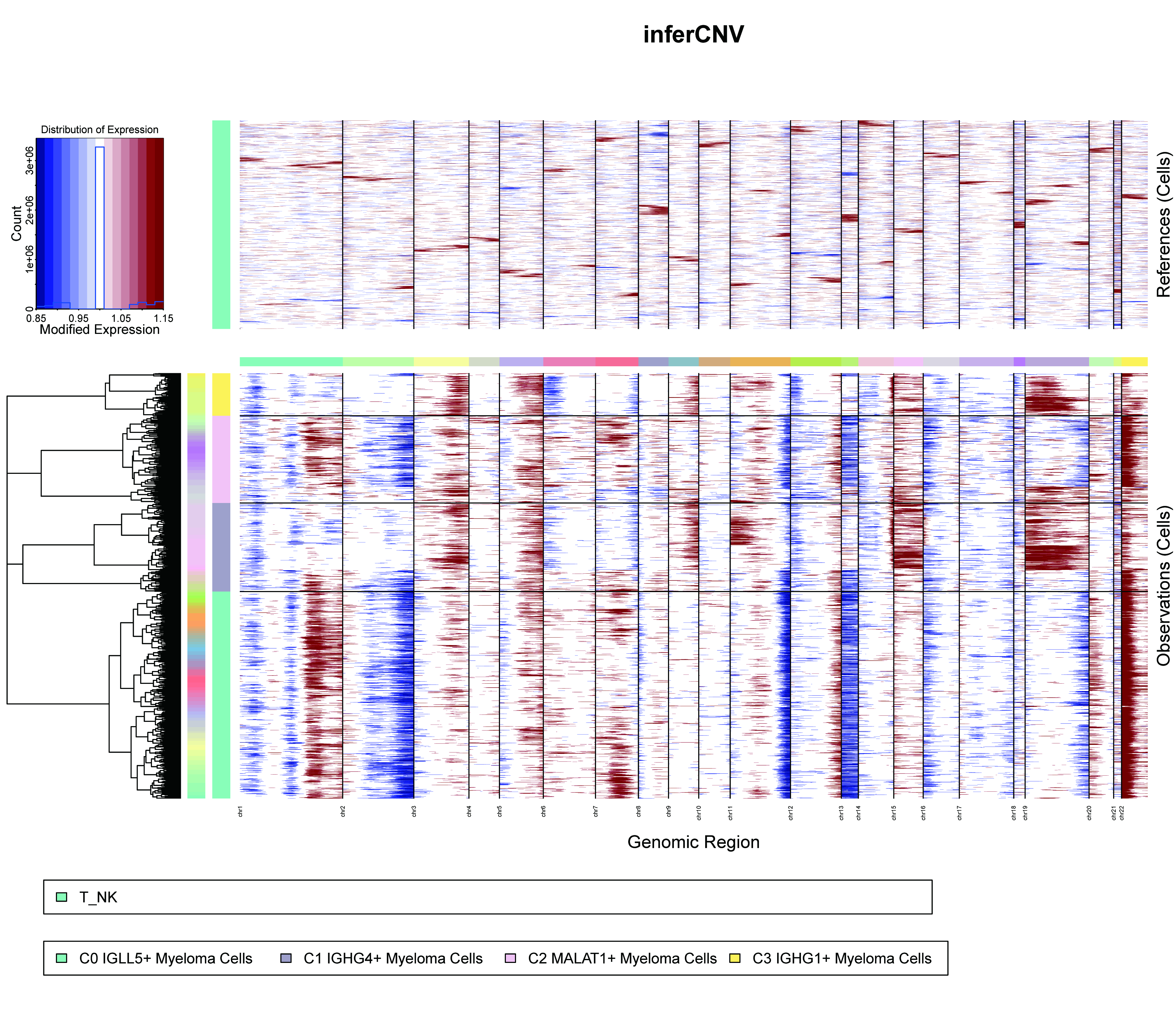

Supplement: Supplementary Figure 1 — InferCNV explored single-cell RNA-seq data from myeloma cells to distinguish the cells that we want to study. [file Image1.tif]
